# Targeting KIFC1 to disrupt centrosome clustering and trigger anaphase catastrophe in small-cell lung cancer

**DOI:** 10.1172/jci.insight.199352

**Published:** 2026-04-08

**Authors:** Natsuki Nakagawa, Minemichi Toda, Akiko Kunita, Masafumi Horie, Masakatsu Tokunaga, Hiroaki Ikushima, Mirei Ka, Takahiro Iida, Manabu Shigeoka, Yukinobu Ito, Takahiro Ando, Kousuke Watanabe, Yasunori Ota, Xi Liu, Ethan Dmitrovsky, Hidenori Kage, Masanori Kawakami

**Affiliations:** 1 Department of Respiratory Medicine and; 2 Next-Generation Precision Medicine Development Laboratory, Graduate School of Medicine, The University of Tokyo, Tokyo, Japan.; 3 Division of Molecular and Genomic Pathology, Department of Pathology, Kobe University Graduate School of Medicine, Kobe, Japan.; 4 Department of Molecular and Cellular Pathology, Graduate School of Medical Sciences, Kanazawa University, Kanazawa, Japan.; 5 Division of Integrative Genomics and; 6 Department of Thoracic Surgery, Graduate School of Medicine, The University of Tokyo, Tokyo, Japan.; 7 Department of Pathology, Research Hospital, The Institute of Medical Science, The University of Tokyo, Tokyo, Japan.; 8 Molecular Pharmacology Program, Frederick National Laboratory for Cancer Research, Frederick, Maryland, USA.

**Keywords:** Cell biology, Oncology, Lung cancer

## Abstract

Supernumerary centrosomes are a hallmark of cancer. To maintain viability, cancer cells cluster these centrosomes during mitosis, enabling bipolar division similar to that of normal cells. Disruption of this centrosome clustering leads to multipolar anaphase and apoptosis (anaphase catastrophe), which selectively eliminates cancer cells harboring supernumerary centrosomes. In this context, because the motor protein KIFC1 contributes to centrosome clustering, we investigated whether targeting of this mechanism through KIFC1 inhibition could be exploited in small-cell lung cancer (SCLC), an aggressive malignancy with limited treatment options and poor prognosis. Through in silico and in vitro analyses, as well as IHC of clinical samples, we found that KIFC1 is overexpressed and that centrosome amplification occurs more frequently in SCLC compared with normal tissues and other cancer types. Pharmacological and genetic inhibition of KIFC1 disrupted the clustering of supernumerary centrosomes, triggered multipolar mitosis, and exerted antineoplastic effects in SCLC cells, with minimal effects on noncancerous cells. These findings were validated and extended in vivo using SCLC xenograft models. Finally, cotargeting KIFC1 and the centrosome duplication regulator PLK4 further enhanced growth suppression in SCLC cells. Together, these results suggest that disrupting centrosome clustering and triggering anaphase catastrophe via KIFC1 inhibition may represent a promising therapeutic strategy for SCLC.

## Abstract 2

By blocking clustering of supernumerary centrosomes, a hallmark of cancer cells, targeting KIFC1 induces multipolar mitosis and lethal anaphase catastrophe in small cell lung cancer.

## Graphical abstract

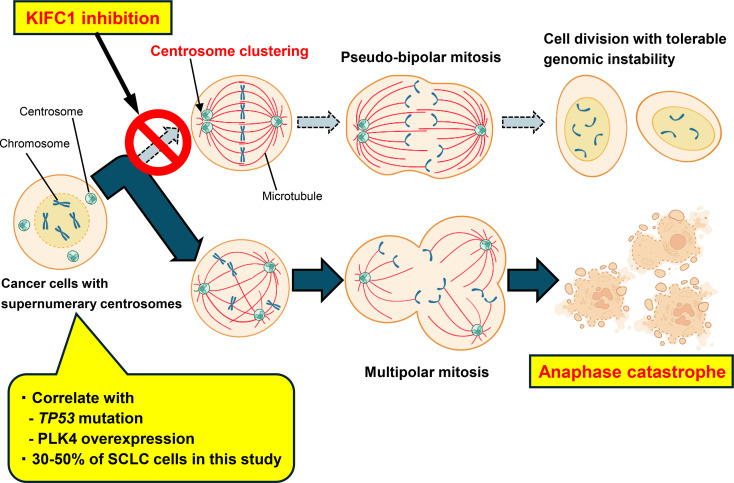

## Introduction

Centrosomes play critical roles serving as spindle poles during cell mitosis. Centrosome duplication, tightly regulated by polo-like kinase 4 (PLK4) (1), occurs only once per cell cycle. In normal cells, centrosome number is strictly controlled, and the presence of exactly 2 centrosomes is essential for proper bipolar spindle formation during mitosis (2, 3). However, cancer cells frequently harbor more than 2 centrosomes (supernumerary centrosomes) (4–10), which are closely associated with genomic instability, a hallmark of cancer (11). Supernumerary centrosomes in cancer cells promote multipolar spindle formation and multipolar mitosis, leading to asymmetric chromosome segregation and aneuploidy. While this contributes to tumor heterogeneity and evolution, excessive chromosome mis-segregation can impair cell viability (4, 12–17). To circumvent these detrimental effects of multipolar mitosis, cancer cells employ mechanisms to suppress multipolar mitosis, the best characterized of which is centrosome clustering (Supplemental Figure 1; supplemental material available online with this article; https://doi.org/10.1172/jci.insight.199352DS1) (15, 18–22). Through this process, supernumerary centrosomes are grouped into 2 functional spindle poles, enabling cancer cells to undergo pseudo-bipolar mitosis and maintain mitotic fidelity (Supplemental Figure 1) (23). This allows cancer cells with supernumerary centrosomes to survive with levels of aneuploidy and genetic heterogeneity that are tolerated (14).

We previously reported that inhibition of cyclin-dependent kinase 2 (CDK2) disrupts the clustering of supernumerary centrosomes, forcing cancer cells to undergo multipolar mitosis, which leads to chromosome mis-segregation and apoptosis (24–27). We termed this pro-apoptotic mechanism “anaphase catastrophe” (28–30). Because supernumerary centrosomes are found almost exclusively in malignant cells, anaphase catastrophe is expected to selectively target cancer cells while sparing normal cells with 2 centrosomes (22, 23). This therapeutic window highlights the potential of anaphase catastrophe as a promising antineoplastic strategy. However, given that CDK2 also plays a role in normal cell cycle progression, its inhibition could consequently affect normal cells (31). Thus, identification of alternative targets capable of selectively inducing anaphase catastrophe is desired.

Kinesin family member C1 (KIFC1/HSET) is a minus end–directed kinesin motor protein of the kinesin 14 family that facilitates spindle assembly and organization during mitosis by sliding and cross-linking adjacent microtubules (32, 33). It is implicated in centrosome clustering as well as chromosome segregation and cell migration in cancer cells (15, 22, 34–37). High KIFC1 expression has been observed in different cancer types (34, 37) and is associated with increased invasion, metastasis, and poor prognosis (34, 35, 38–41). Previous studies demonstrated in vitro antiproliferative effects following KIFC1 inhibition in hepatocellular, pancreatic, prostate, and non–small cell lung cancer cells (38, 42–46). Notably, while KIFC1 plays an essential role in centrosome clustering in cancer cells, prior studies suggest that its inhibition has relatively limited effects on the survival of noncancerous cells (47–49).

Small-cell lung cancer (SCLC) is among the most clinically aggressive and fatal solid tumors and accounts for about 15% of lung cancer cases (50). Although SCLC initially responds to cytotoxic chemotherapy, most patients eventually experience recurrence and metastasis, with a dismal prognosis: the median survival is only 10–13 months, and the 5-year survival rate is about 2%. While immune checkpoint inhibitors were added to the SCLC treatment regimen, their clinical efficacy remains limited (51, 52). Consequently, there is a need to develop innovative therapeutic strategies for SCLC. Notably, SCLC is characterized by profound genomic instability and near-universal inactivation of *TP53* and *RB1* (50, 53). Loss of these tumor suppressors disrupts cell cycle checkpoints and the DNA damage response, making further perturbations to mitotic control potentially lethal to SCLC cells (54). Additionally, p53 inactivation has been linked to centrosome amplification (55, 56). Based on these observations, we hypothesized that SCLC cells frequently harbor supernumerary centrosomes and are particularly susceptible to anaphase catastrophe, a distinct form of mitotic failure that results from disrupted centrosome clustering.

In this study, we investigated the engagement of anaphase catastrophe via KIFC1 inhibition as a promising therapeutic strategy for SCLC. In silico analyses using publicly available databases, in vitro experiments with cell lines, and IHC of clinical specimens revealed KIFC1 overexpression and a high frequency of supernumerary centrosomes in SCLC as compared with normal tissues and other cancer types. Subsequently, we systematically evaluated the effects of KIFC1 inhibition in SCLC cells. The results demonstrated that KIFC1 inhibition disrupted centrosome clustering and induced anaphase catastrophe in SCLC cells. These effects were not observed in noncancerous bronchial cells, which rarely exhibit centrosome amplification. These in vitro findings were confirmed and extended to the in vivo setting using human SCLC xenograft models. Finally, building on the role of PLK4 in regulating centrosome duplication, we also found that combining KIFC1 inhibition with PLK4 targeting further enhanced growth suppression in SCLC cells. Collectively, our findings indicate that disrupting centrosome clustering and triggering anaphase catastrophe by KIFC1 inhibition represents a promising strategy to combat SCLC, a highly lethal malignancy.

## Results

### KIFC1 overexpression and centrosome amplification in SCLC.

**Figure 1:**
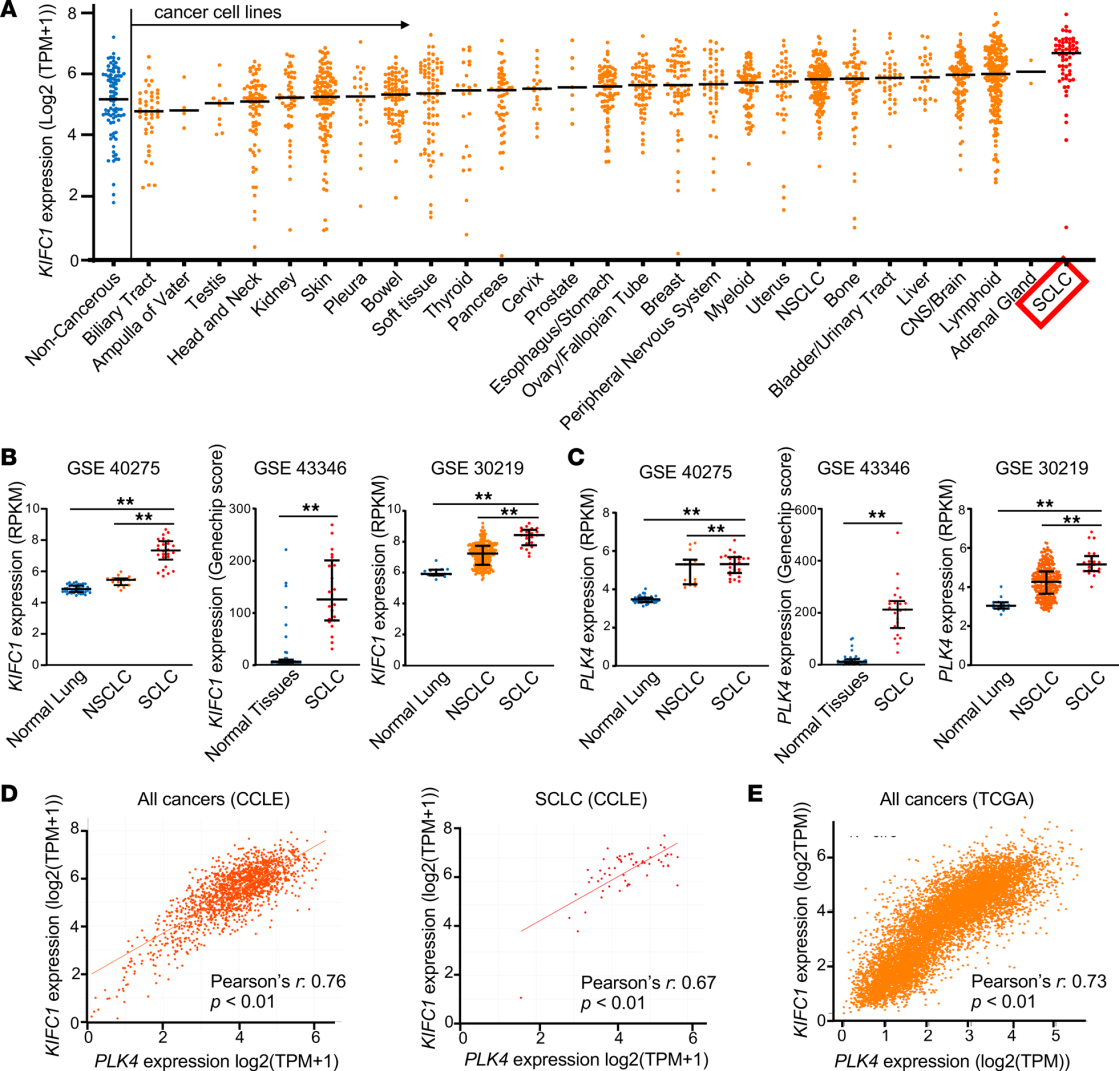
In silico analysis of KIFC1 and PLK4 expression in SCLC and other cancer types. (**A**) *KIFC1* mRNA expression levels were compared among various cancer and noncancerous cell lines using the CCLE database. Bars indicate median values. TPM, transcripts per million. (**B** and **C**) *KIFC1* (**B**) and *PLK4* (**C**) mRNA expression levels in clinical samples were compared among SCLC, NSCLC, and normal tissues using datasets from the GEO database. GSE40275 includes 26 SCLC, 15 NSCLC, and 43 normal lung tissue samples. GSE43346 includes 23 SCLC and 43 normal tissue samples. GSE30219 includes 21 SCLC, 268 NSCLC, and 14 normal lung tissue samples. Bars represent median values and interquartile range. Two-sided *t* tests were performed with multiple comparisons adjusted using Tukey’s method. **P < 0.01. RPKM, Reads Per Kilobase of exon per Million mapped reads. (**D**) Correlation of *KIFC1* and *PLK4* mRNA expression levels was analyzed in all cancer-type cell lines (Pearson’s *r* = 0.76, *P* < 0.01) and SCLC cell lines (Pearson’s *r* = 0.67, *P* < 0.01) using the CCLE database. (**E**) Correlation between *KIFC1* and *PLK4* mRNA expression in clinical samples was analyzed using the TCGA database (Pearson’s *r* = 0.73, *P* < 0.01).

We first analyzed mRNA expression levels of *KIFC1* in SCLC using in silico databases. The analysis of the Cancer Cell Line Encyclopedia (CCLE) database revealed that KIFC1 expression in SCLC cell lines was the highest among all cancer types and noncancerous cells (Figure 1A). When *KIFC1* expression across the four SCLC transcriptional subtypes (SCLC-A, -N, -P, and -I, defined by dominant expression of ASCL1, NEUROD1, POU2F3, and an inflamed gene signature, respectively) was examined, no significant difference was observed (Supplemental Figure 2). To extend this analysis to patient-derived transcriptomic data, we next analyzed clinical gene expression datasets in the Gene Expression Omnibus (GEO) database, which showed that *KIFC1* expression was substantially higher in SCLC than in normal tissues and non–small cell lung cancer (NSCLC) (Figure 1B). We also examined the expression of PLK4, a key regulator of centrosome duplication. The analysis of the GEO database revealed that *PLK4* expression was substantially elevated in SCLC as compared with normal tissues and NSCLC (Figure 1C). Since PLK4 overexpression is associated with centrosome amplification (57, 58), these findings suggest a high prevalence of supernumerary centrosomes in SCLC. Furthermore, strong positive correlation was found to exist between *KIFC1* and *PLK4* expression across cancer cell lines in the CCLE database (Figure 1D), and in clinical cancer samples from The Cancer Genome Atlas (TCGA) database (Figure 1E), indicating a potential link between KIFC1 and supernumerary centrosomes.

**Figure 2:**
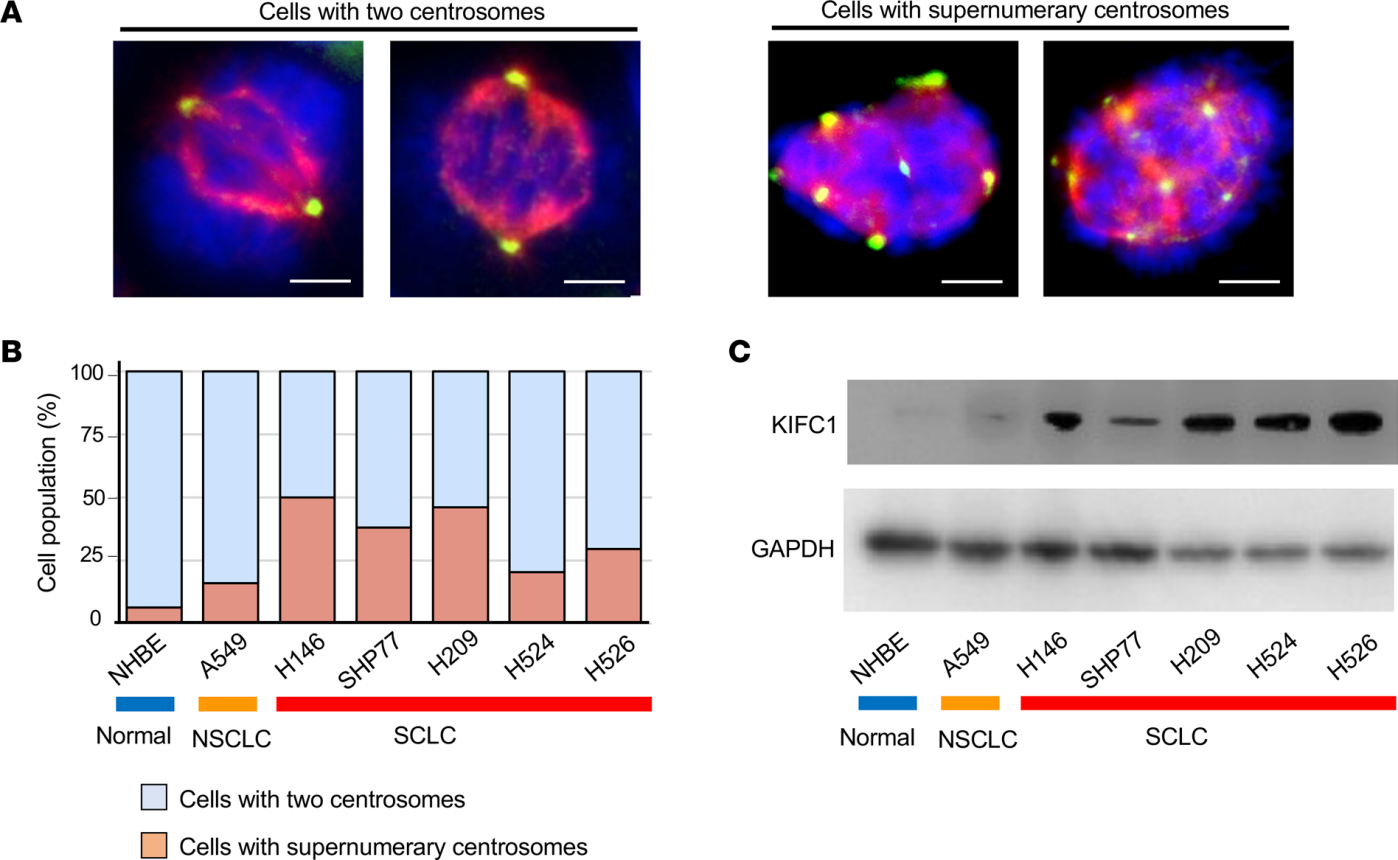
Frequent supernumerary centrosomes and elevated KIFC1 protein levels in SCLC cells. (**A**) Representative immunofluorescence images of SCLC cells with or without supernumerary centrosomes. The blue signal is Hoechst 33342 staining (DNA), the red signal is α-tubulin staining (spindles), and the green signal is pericentrin staining (centrosomes). Scale bars: 5 μm. Data are representative of 3 independent experiments. (**B**) Proportions of cells with or without supernumerary centrosomes in SCLC, NSCLC, and noncancerous cells. (**C**) KIFC1 protein expression levels were assessed by immunoblotting in SCLC, NSCLC, and noncancerous cells.

To validate these in silico findings, we examined the features of centrosomes in cell lines. Immunofluorescence staining for pericentrin, a component of the pericentriolar material and a centrosome marker, revealed a high frequency of supernumerary centrosomes in SCLC cells (Figure 2, A and B). Specifically, 25%–50% of mitotic SCLC cells harbored supernumerary centrosomes, as compared with only 5% of noncancerous normal human bronchial epithelial (NHBE) cells and 15% of NSCLC A549 cells (Figure 2B). We next assessed KIFC1 protein expression levels by immunoblotting, which showed higher expression profiles in SCLC cells than in NHBE and NSCLC cells (Figure 2C), consistent with the in silico mRNA results (Figure 1, A and B).

**Figure 3:**
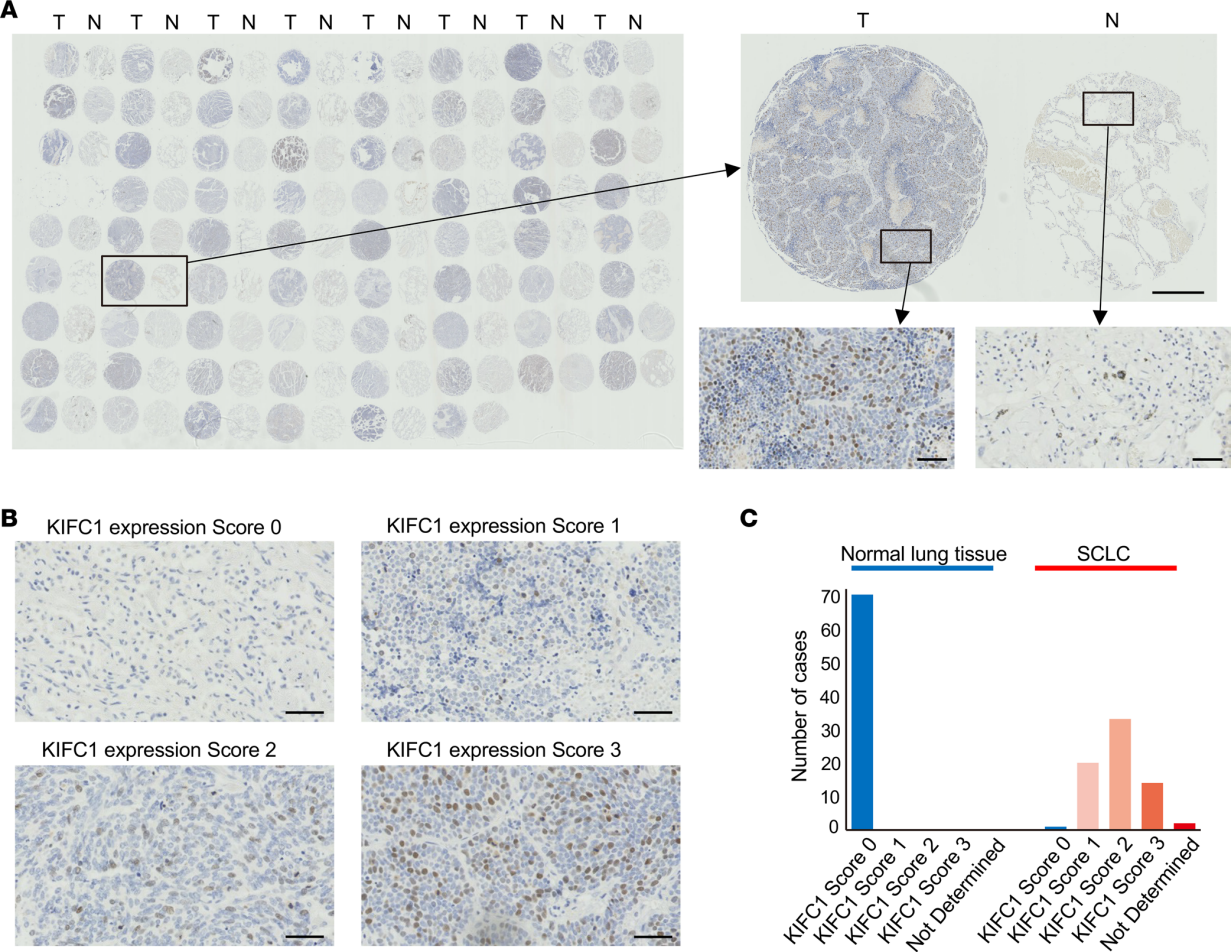
IHC analysis of KIFC1 protein expression in paired SCLC and adjacent normal lung tissues. (**A**–**C**) IHC for KIFC1 on a TMA consisting of 70 paired SCLC and adjacent normal lung tissues. (**A**) Representative images comparing KIFC1 immunostaining in SCLC (T) versus adjacent normal lung tissue (N) including a whole-slide TMA overview and magnified views at lower and higher magnification. Scale bars: 500 μm (lower magnification) and 50 μm (higher magnification). (**B**) Representative images for semiquantitative scoring of KIFC1 expression based on the percentage of positively stained cells: score 0 (<1%), score 1 (1%–10%), score 2 (10%–30%), and score 3 (30%–100%). Scale bars: 50 μm. (**C**) Distribution of KIFC1 expression scores in SCLC versus normal lung tissues.

**Figure 4:**
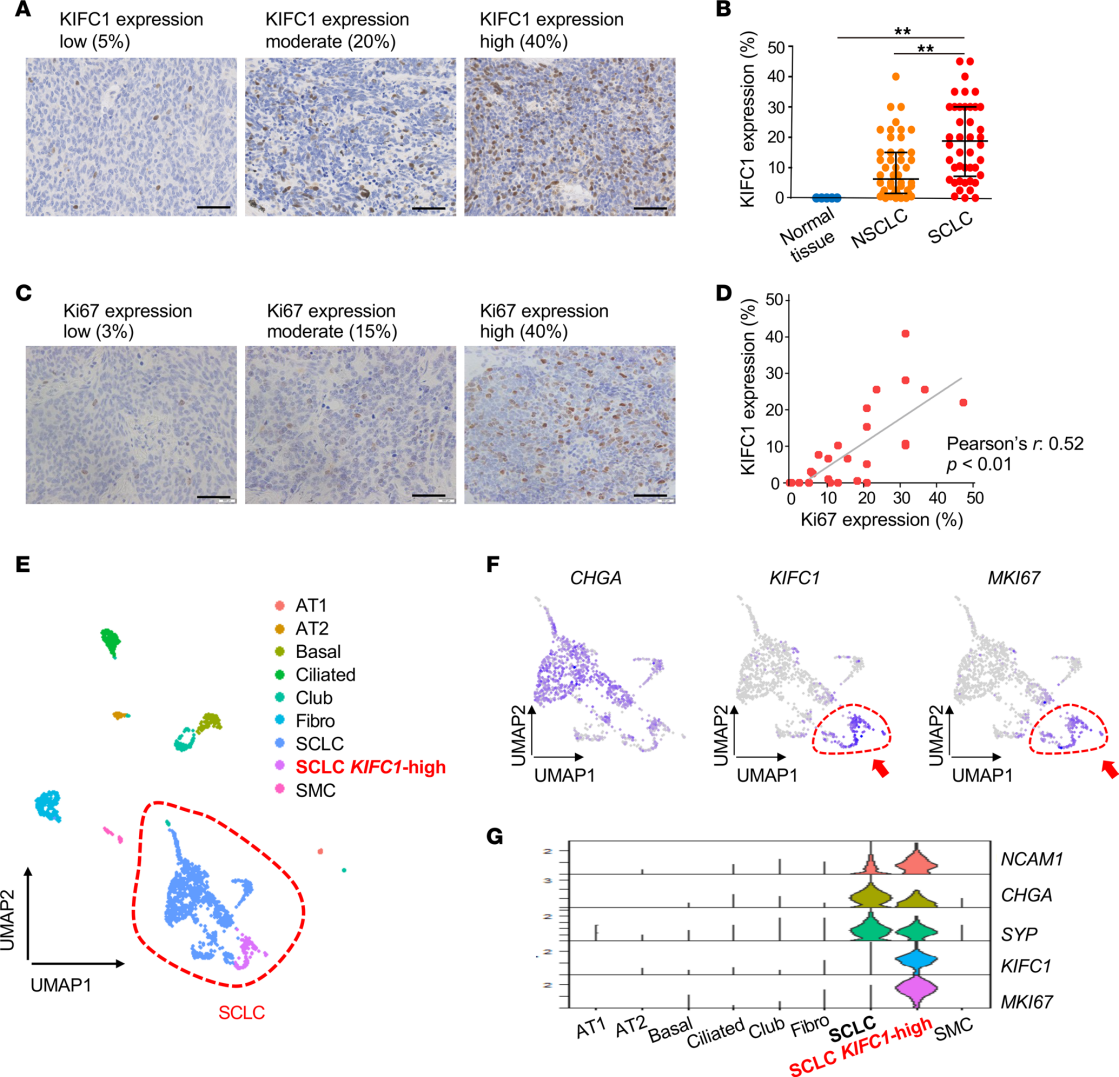
IHC analysis of KIFC1 protein expression in SCLC clinical samples using an independent in-house TMA and in silico scRNA-seq analysis. (**A**–**E**) IHC for KIFC1 on a separate TMA consisting of 47 SCLC, 47 NSCLC, and 5 normal lung tissue samples. (**A** and **B**) The percentage of KIFC1-positive cells was quantified. Representative images of low, moderate, and high levels of KIFC1 expression are shown in **A**. Scale bars: 50 μm. (**B**) KIFC1 expression levels were compared across SCLC, NSCLC, and normal lung tissues. Each dot represents a single case. **P < 0.01 (Tukey’s test). Bars represent median values and interquartile range. (**C** and **D**) Ki67 expression levels were evaluated on the same TMA. Representative images of low, moderate, and high Ki67 expression are shown in **C**. Scale bars: 50 μm. (**D**) Correlation between Ki67 and KIFC1 expression in SCLC cases was assessed (Pearson’s *r* = 0.52, *P* < 0.01). Each dot represents a single case. (**E**–**G**) scRNA-seq analysis of SCLC (GSE164404). (**E**) UMAP clustering of single-cell transcriptomes from SCLC. (**F**) Feature plots showing the expression of the neuroendocrine marker (*CHGA*), *KIFC1*, and *MKI67* in SCLC. (**G**) Violin plots displaying the expression of neuroendocrine markers (*CHGA*, *NCAM1*, *SYP*), *KIFC1*, and *MKI67* across different cell types.

We further evaluated KIFC1 expression in SCLC clinical samples by IHC using 2 independent tissue microarrays (TMAs) of specimens isolated from SCLC patients. Analysis of the first TMA, consisting of 70 paired SCLC and adjacent normal lung tissues, revealed that KIFC1 was highly expressed in most SCLC tissues, whereas it was typically undetected in adjacent normal tissues (Figure 3, A–C). To independently confirm and extend these findings, we analyzed a separate TMA from our institution that displayed 47 SCLC, 47 NSCLC, and 5 normal lung tissues, with gene expression data from these specimens available. Patient characteristics for this cohort are summarized in Supplemental Table 1. KIFC1 expression was significantly higher in SCLC samples than in the examined NSCLC and normal lung samples (Figure 4, A and B), validating the results from the first TMA shown above (Figure 3, A–C). When analyzed by transcriptional subtype, KIFC1 expression did not differ significantly among SCLC-A, -N, -P, and -I (Supplemental Figure 3A). However, when SCLC-A and SCLC-N, which both represent neuroendocrine-high subtypes, were combined and compared with SCLC-P/I, KIFC1 expression was significantly higher in the former group (Supplemental Figure 3B). Additionally, it was revealed that KIFC1 expression positively correlated with Ki67 levels, a marker of cellular proliferation (Figure 4, C and D). Consistent with its link to proliferative activity, survival analysis in our institutional TMA cohort showed a trend toward shorter overall survival with increasing KIFC1 expression (*P* = 0.021, log-rank test for trend; *P* = 0.053 for high vs. low groups; Supplemental Figure 4), suggesting that elevated KIFC1 expression may be associated with poor prognosis in SCLC. This correlation was further supported by the analyses of publicly available single-cell RNA sequencing (scRNA-seq) datasets (Figure 4, E–G). In silico analysis of scRNA-seq data identified a distinct subpopulation of SCLC cells with high *KIFC1* expression, which overlapped with the highly proliferative Ki67-positive subpopulation (Figure 4, E–G). Characterization of transcriptomic features in these *KIFC1*-high cells revealed upregulation of multiple regulators of centrosome organization and mitotic control, including *PLK4*, *AURKA*, *AURKB*, *TTK*, *PLK1*, and *CHEK2* (Supplemental Table 2 and Supplemental Figure 5A). Pathway analysis further indicated activation of cell cycle–, mitosis-, and checkpoint-related programs (Supplemental Figure 5B), consistent with a highly proliferative phenotype.

### Antineoplastic effects after KIFC1 inhibition in SCLC cells.

**Figure 5:**
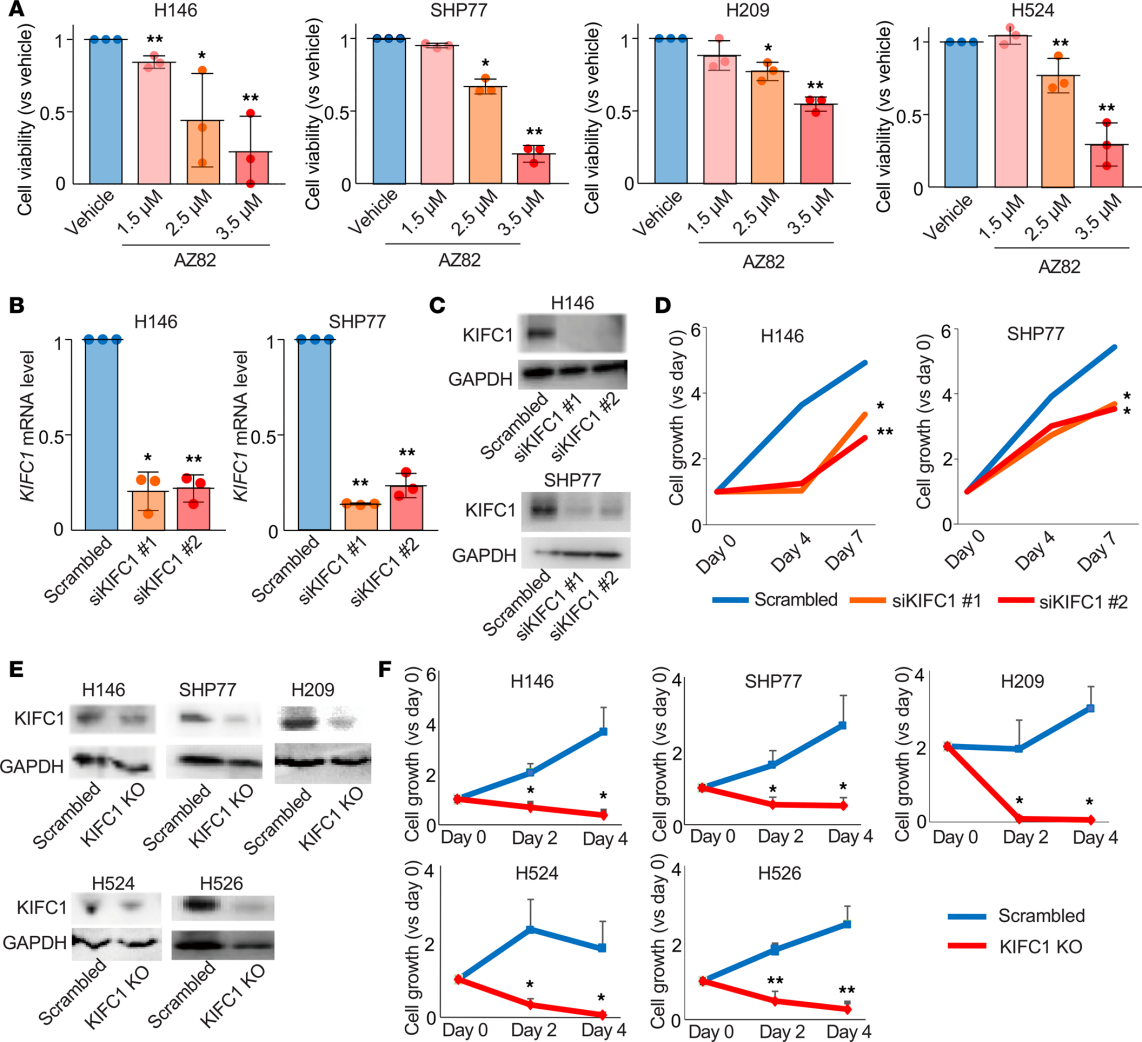
Antiproliferative effects of KIFC1 inhibition in SCLC cells. (**A**) Dose-dependent effects of AZ82 on cell viability in multiple SCLC cell lines are demonstrated. (**B** and **C**) Knockdown of KIFC1 expression using siRNAs was confirmed at the mRNA level by RT-qPCR (**B**) and at the protein level by immunoblotting (**C**). (**D**) Cell proliferation curves of SCLC cells following KIFC1 knockdown. (**E**) Depletion of KIFC1 using the CRISPR/Cas9 system was confirmed by immunoblotting. (**F**) Cell proliferation curves of SCLC cell lines following KIFC1 knockout. Error bars represent standard deviation. *P* values were calculated using 2-sided *t* tests with multiple comparisons adjusted using Dunnett’s method. *P < 0.05; **P < 0.01.

To investigate the biological function of KIFC1 in SCLC, we inhibited KIFC1 in multiple SCLC cell lines using 3 approaches: pharmacological inhibition with AZ82, a specific KIFC1 inhibitor, and genetic inhibition by siRNAs and by the CRISPR/Cas9 system. Treatment with AZ82 reduced the viability of SCLC cell lines in a dose-dependent manner (Figure 5A). Knockdown of KIFC1 using siRNAs was confirmed by real-time quantitative reverse transcription PCR (RT-qPCR) and immunoblotting (Figure 5, B and C). Following KIFC1 knockdown, cell proliferation was significantly suppressed in SCLC cells (Figure 5D). Similarly, CRISPR/Cas9–mediated depletion of KIFC1, verified by immunoblotting (Figure 5E), significantly inhibited SCLC cell proliferation (Figure 5F). These findings demonstrate that KIFC1 inhibition suppressed the growth of SCLC cells. Because these experiments were performed using pooled cell populations rather than single-cell clones, a small amount of residual KIFC1 protein was detected after CRISPR/Cas9–mediated depletion, reflecting a partial but substantial reduction in KIFC1 protein levels.

**Figure 6:**
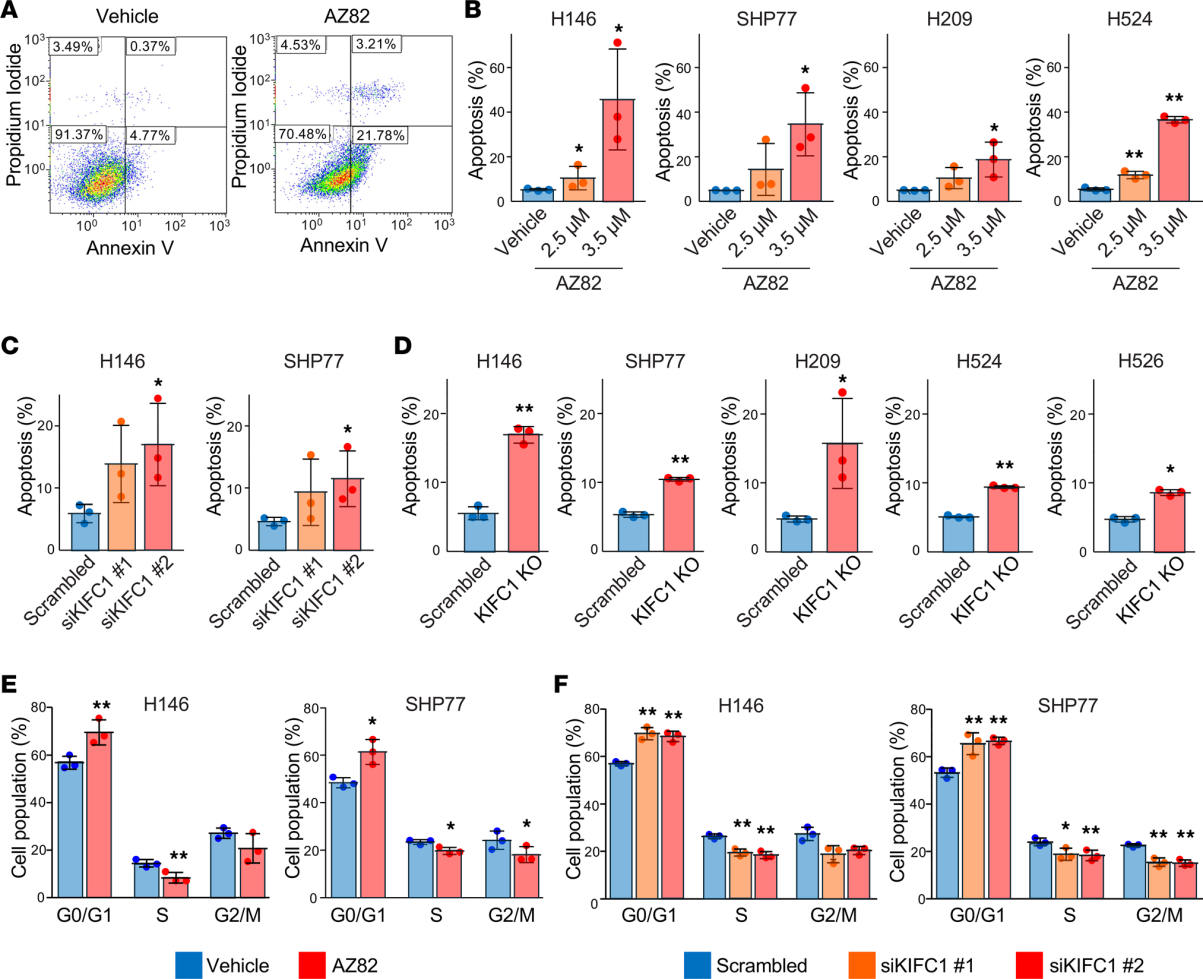
Apoptosis induction and cell cycle arrest in SCLC cells following KIFC1 inhibition. (**A**) Representative flow cytometric dot plots showing annexin V and propidium iodide double staining to assess apoptosis following KIFC1 inhibition. Data are representative of 3 independent experiments. (**B**) Percentages of apoptotic cells after AZ82 treatment in SCLC cells. (**C**) Percentages of apoptotic cells following KIFC1 knockdown by siRNAs in SCLC cells. (**D**) Percentages of apoptotic cells following KIFC1 knockout by the CRISPR/Cas9 system in SCLC cells. (**E**) Cell cycle distribution in SCLC cells after AZ82 treatment. (**F**) Cell cycle distribution in SCLC cells following KIFC1 knockdown by siRNAs. Error bars represent standard deviation. *P* values were calculated using 2-sided *t* tests with multiple comparisons adjusted using Dunnett’s method. *P < 0.05; **P < 0.01.

We next evaluated the effects of KIFC1 inhibition on apoptosis. Representative flow cytometry images of annexin V/propidium iodide double staining to assess apoptosis after treatment with vehicle or AZ82 are shown in Figure 6A. AZ82 treatment augmented apoptosis in a dose-dependent manner in the examined SCLC cells (Figure 6B). Apoptosis induction was confirmed by genetic inhibition of KIFC1 using siRNAs (Figure 6C) and the CRISPR/Cas9 system in SCLC cells (Figure 6D). To assess whether apoptosis functionally contributes to the growth inhibition caused by KIFC1 blockade, SCLC cells were treated with AZ82 in the presence or absence of the pan-caspase inhibitor Q-VD-OPh. A modest but reproducible rescue of viability was observed (Supplemental Figure 6), indicating that caspase-dependent apoptosis contributes to, but does not fully account for, the growth suppression induced by KIFC1 inhibition.

Finally, we investigated the effects of KIFC1 inhibition on cell cycle progression after synchronizing cells. KIFC1 inhibition induced G_1_-phase arrest in SCLC cells, either through AZ82 treatment (Figure 6E) or by siRNA-mediated knockdown (Figure 6F).

### Anaphase catastrophe via KIFC1 inhibition in SCLC cells.

**Figure 7:**
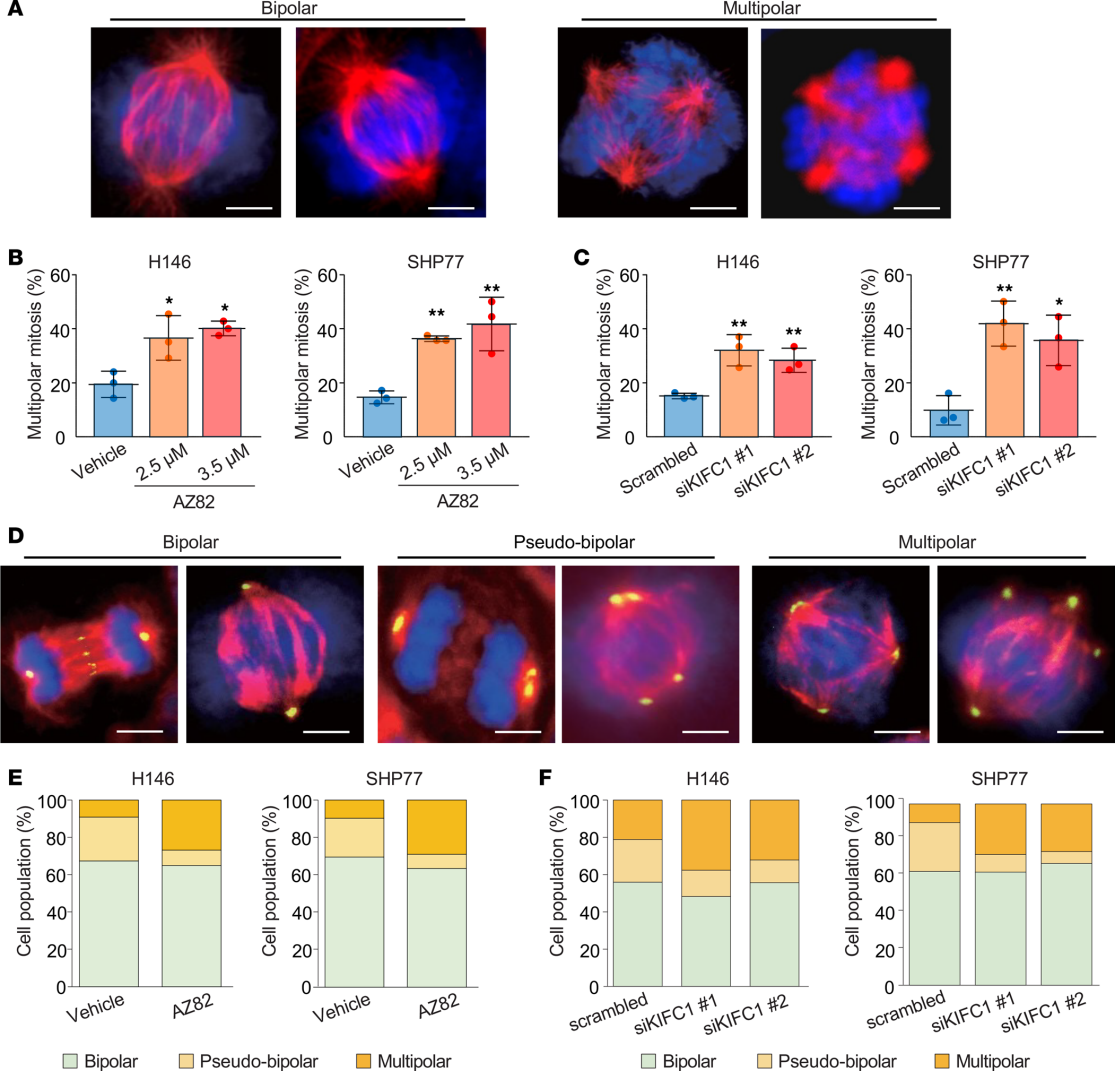
Induction of anaphase catastrophe in SCLC cells following KIFC1 inhibition. (**A**) Representative immunofluorescence images of SCLC cells undergoing bipolar and multipolar anaphase. The blue signal is Hoechst 33342 staining (DNA), and the red signal is α-tubulin staining (spindles). Scale bars: 5 μm. Data are representative of 3 independent experiments. (**B** and **C**) Percentages of SCLC cells undergoing multipolar anaphase after AZ82 treatment (**B**) and after KIFC1 knockdown using siRNAs (**C**). Error bars represent standard deviation. *P* values were calculated using Dunnett’s method. *P < 0.05; **P < 0.01. (**D**) Representative immunofluorescence images showing 3 mitotic phenotypes in SCLC cells: bipolar cells with 2 centrosomes and normal bipolar spindles (left panels), pseudo-bipolar cells with supernumerary centrosomes clustered into 2 poles forming bipolar spindles (middle panels), and multipolar cells with unclustered supernumerary centrosomes forming multipolar spindles (right panels). The blue signal is Hoechst 33342 staining (DNA), the red signal is α-tubulin staining (spindles), and the green signal is pericentrin staining (centrosomes). Scale bars: 5 μm. Data are representative of 3 independent experiments. (**E** and **F**) Distribution of bipolar, pseudo-bipolar, and multipolar SCLC cells after AZ82 treatment (**E**) and after KIFC1 knockdown using siRNAs (**F**).

To determine the mechanism underlying the antineoplastic effects of KIFC1 inhibition, we examined the mitotic status of SCLC cells by staining for α-tubulin and DNA. In addition to normal bipolar mitotic cells, multipolar mitotic cells were detected (Figure 7A). Treatment with AZ82 and KIFC1 knockdown using siRNAs markedly increased the proportion of cells undergoing multipolar anaphase (Figure 7, B and C). We next performed costaining with pericentrin to assess centrosome features. Based on spindle and centrosome configurations, these cells were categorized into 3 types: normal bipolar cells with 2 centrosomes and bipolar spindles, pseudo-bipolar cells with supernumerary centrosomes clustered into 2 poles and bipolar spindle formation, and multipolar cells with supernumerary centrosomes that failed to cluster, resulting in multipolar spindle formation (Figure 7D and Supplemental Figure 1). Notably, inhibition of KIFC1 either pharmacologically or genetically reduced the proportion of pseudo-bipolar cells and increased the proportion of multipolar cells (Figure 7, E and F, and Supplemental Figures 7 and 8), while the total proportion of cells with supernumerary centrosomes (pseudo-bipolar and multipolar cells) was not appreciably altered (Figure 7, E and F, and Supplemental Figure 7). These results indicate that KIFC1 inhibition disrupts the clustering of preexisting supernumerary centrosomes, thereby promoting multipolar mitosis and exerting antineoplastic effects, i.e., inducing anaphase catastrophe, in SCLC cells.

### Noncancerous cells show minimal sensitivity to KIFC1 inhibition.

Anaphase catastrophe is expected to preferentially affect cancer cells harboring supernumerary centrosomes, while sparing normal cells with 2 centrosomes. To evaluate this selectivity, we examined the effects of KIFC1 inhibition in noncancerous bronchial epithelial cells, including NHBE cells and the immortalized but nontransformed bronchial epithelial cell line BEAS-2B. Treatment with AZ82 did not appreciably inhibit the growth of either NHBE or BEAS-2B cells (Supplemental Figure 9A) at concentrations that substantially reduced proliferation in SCLC cells (Figure 5A). We further silenced KIFC1 genetically using siRNAs transfected into these noncancerous cells. Although effective knockdown of KIFC1 was confirmed at both mRNA and protein levels (Supplemental Figure 9, B and C), cell proliferation remained unaffected (Supplemental Figure 9D). In addition, the mitotic analysis revealed that KIFC1 knockdown did not induce multipolar mitosis in either NHBE or BEAS-2B cells (Supplemental Figure 9E). Collectively, these results indicate that, under experimental conditions tested, KIFC1 inhibition exerts minimal effects on proliferation and mitotic spindle integrity in noncancerous bronchial epithelial cells, in contrast to its pronounced antineoplastic effects in SCLC cells.

### Transcriptomic changes after KIFC1 inhibition in SCLC cells.

**Figure 8:**
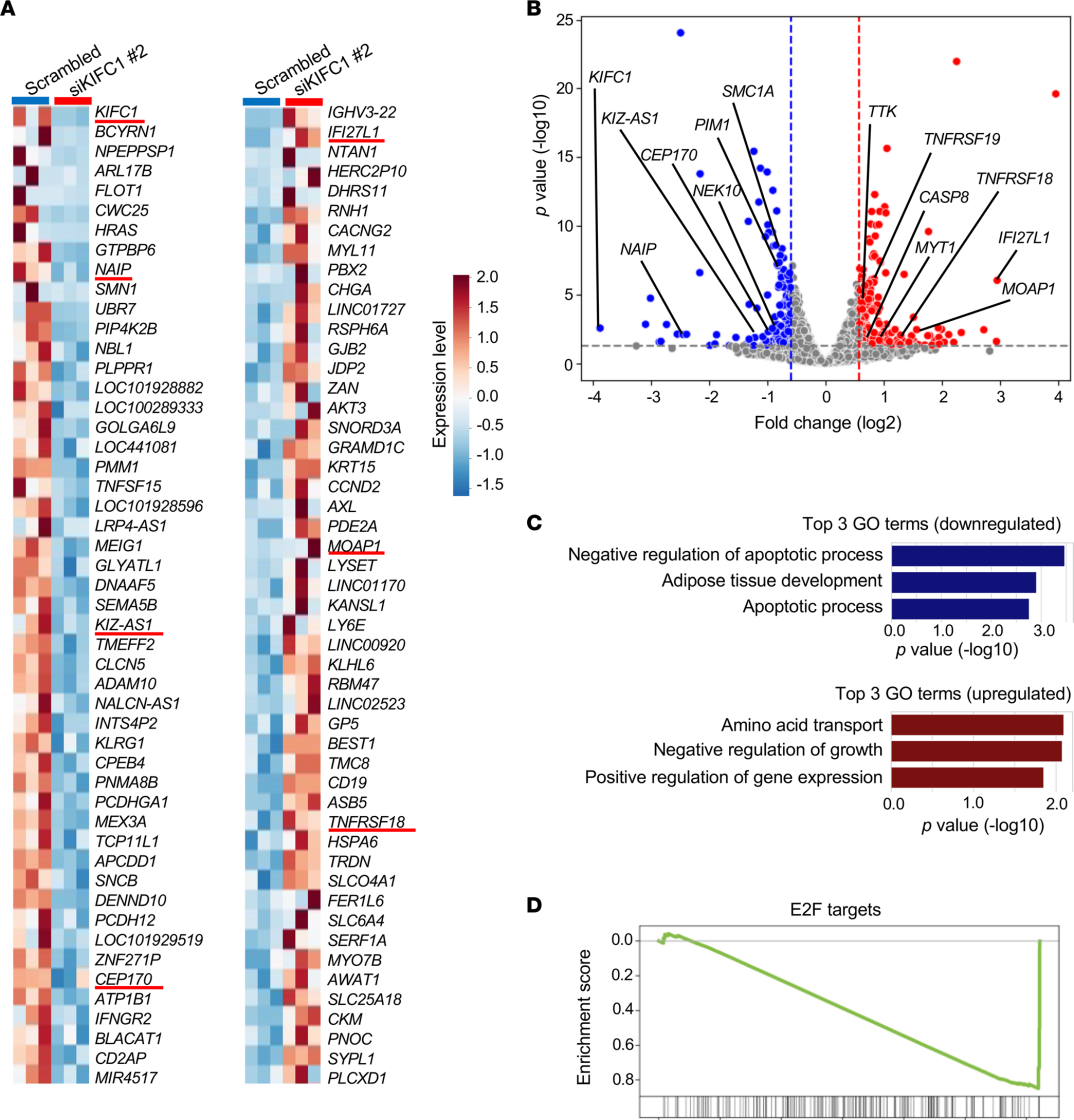
Transcriptomic profiles of SCLC cells following KIFC1 inhibition. (**A**) Heatmaps showing the top 50 differentially expressed genes in H146 cells after KIFC1 knockdown (left, downregulated genes; right, upregulated genes). (**B**) Volcano plots of differentially expressed genes after KIFC1 knockdown. Thresholds were set at *P* < 0.05 and absolute fold change > 1.5. Blue and red dots indicate downregulated and upregulated genes meeting these thresholds, respectively. Statistical significance was assessed using Wilcoxon’s rank-sum test (individual group comparison). (**C**) Top 3 downregulated and upregulated pathways identified by Gene Ontology (GO) analysis. (**D**) GSEA of downregulated gene sets following KIFC1 knockdown.

To elucidate the molecular mechanisms underlying the antineoplastic effects and anaphase catastrophe after KIFC1 inhibition, we performed RNA sequencing and analyzed transcriptomic changes following KIFC1 knockdown in H146 SCLC cells. The top 50 differentially expressed genes are shown in Figure 8A. Using cutoffs of *P* < 0.05 and fold change > 1.5 or < –1.5, we identified 148 downregulated and 177 upregulated genes (Figure 8B and Supplemental Table 3). Notably, several genes involved in centrosome function, clustering, and faithful chromosome segregation, such as *KIZ-AS1*, *CEP170*, and *NEK10*, were significantly downregulated (Figure 8, A and B). Conversely, the upregulated genes included those associated with apoptosis and cell cycle checkpoint pathways (Figure 8, A and B). Gene Ontology analysis revealed downregulation of genes that negatively regulate apoptosis and upregulation of genes that negatively regulate growth following KIFC1 knockdown (Figure 8C). Furthermore, GSEA identified E2F targets, which play major roles during the G_1_/S transition, as significantly downregulated (Figure 8D). Together, these transcriptomic profiles indicate that disruption of centrosome clustering by KIFC1 inhibition activates transcriptional programs linked to mitotic stress, checkpoint activation, and apoptotic signaling. This provides molecular-level insight into how centrosome dysregulation translates into growth arrest and cell death in SCLC.

### In vivo effects after KIFC1 inhibition.

In vivo effects of KIFC1 inhibition were examined using AZ82 in human SCLC cell line–derived xenograft models. We conducted a preliminary study to determine the optimal dosage of AZ82. Intraperitoneal (i.p.) administration of AZ82 at doses of 15 μg/g or higher caused either death or substantial weight loss in the treated mice. Doses of 12.5 μg/g or lower were well tolerated with no weight loss observed during 2 weeks of test administration. Based on these findings, 12.5 μg/g i.p. was selected as the treatment dose for subsequent experiments.

**Figure 9:**
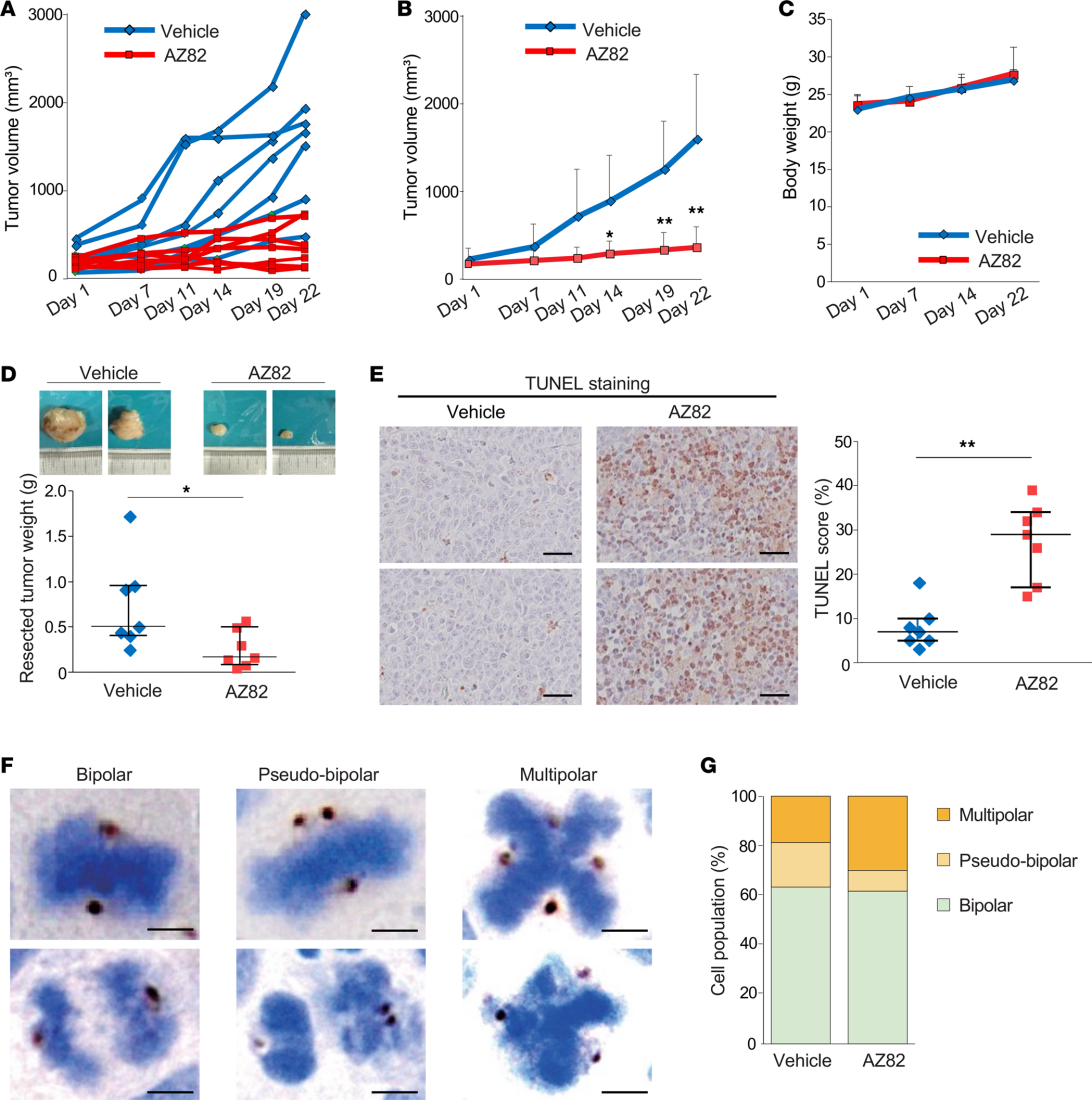
In vivo antitumor effects of AZ82 treatment in SCLC. (**A** and **B**) Comparison of tumor growth in H146-derived xenograft model treated with vehicle or AZ82. Spider plots showing tumor volume for each mouse are shown in **A**, and average tumor volumes over time are shown in **B**. Error bars represent standard deviation. *P* values were calculated using 2-sided *t* tests. *P < 0.05; **P < 0.01. (**C**) Body weights of mice during vehicle or AZ82 treatment. (**D**) Comparison of resected tumor weights after vehicle or AZ82 treatment. Top panels show representative photographs of resected tumors from each group. Each dot represents a single mouse. Bars indicate median values and interquartile range. *P* values were calculated using 2-sided *t* tests. *P < 0.05. (**E**) Quantification of apoptotic cells in resected tumors using TUNEL staining. Left panels show representative images of TUNEL-negative and TUNEL-positive samples from each group. Scale bars: 50 μm. Right panel shows the percentage of TUNEL-positive cells per mouse. Each dot represents a single mouse. Bars indicate median values and interquartile range. *P* values were calculated using a 2-sided *t* test. **P < 0.01. (**F** and **G**) Mitotic status and centrosome organization in resected tumors, evaluated by pericentrin staining with hematoxylin counterstaining. (**F**) Representative pericentrin-immunostained images showing 3 mitotic phenotypes in SCLC cells: bipolar cells with 2 centrosomes and normal bipolar spindles (left panels), pseudo-bipolar cells with clustered supernumerary centrosomes forming bipolar spindles (middle panels), and multipolar cells with unclustered supernumerary centrosomes forming multipolar spindles (right panels). Scale bars: 5 μm. (**G**) Percentages of bipolar, pseudo-bipolar, and multipolar mitotic cells in resected tumors are shown. Representative images shown in **D**–**F** were selected from tumors collected from all mice in each treatment group.

H146 cells were subcutaneously injected into nude mice, followed by administration of either AZ82 (12.5 μg/g) or vehicle 4 times per week for 3 weeks. AZ82 treatment substantially suppressed tumor growth as compared with vehicle control (Figure 9, A and B). No appreciable body weight loss or behavioral changes were observed during treatment, indicating clinical tolerability of AZ82 in mice at this dosage (Figure 9C). Resected tumor weights after completion of treatment were significantly lower in the AZ82-treated group as compared with the vehicle-treated mice (Figure 9D). In addition, IHC staining for TUNEL revealed a higher apoptotic index in the AZ82-treated tumors, indicating enhanced apoptosis after AZ82 treatment (Figure 9E).

To investigate the in vivo effects of KIFC1 inhibition on mitosis and centrosome status, we stained resected tumors for pericentrin and counterstained with hematoxylin. As seen in vitro (Figure 7D), 3 types of mitotic cells were identified: normal bipolar, pseudo-bipolar (with clustered supernumerary centrosomes), and multipolar cells (with unclustered supernumerary centrosomes) (Figure 9F). Consistent with our in vitro findings, the proportion of pseudo-bipolar mitotic figures were reduced while multipolar mitoses were increased in the AZ82-treated tumors (Figure 9G), suggesting that KIFC1 inhibition disrupted centrosome clustering and induced anaphase catastrophe.

To further support the reproducibility of the in vivo antitumor effects of KIFC1 inhibition, we performed an additional xenograft study with the SCLC cell line SHP77. Using the same AZ82 dosing regimen as in the H146 model, AZ82 treatment similarly suppressed tumor growth in SHP77 xenografts without inducing appreciable body weight loss (Supplemental Figure 10). These findings demonstrate that the antitumor efficacy of KIFC1 inhibition in vivo can be reproduced in an additional SCLC xenograft model.

Taken together, the effects of KIFC1 inhibition on SCLC observed in vitro were largely recapitulated and reinforced in the in vivo xenograft models.

### Combined effects of KIFC1 inhibition and PLK4 modulation.

CFI-40095 is a selective modulator of PLK4 (59). At low concentrations, CFI-400945 interferes with the negative autoregulation of PLK4 and leads to accumulation of active PLK4, thereby inducing centrosome amplification and generating supernumerary centrosomes (59, 60).

**Figure 10:**
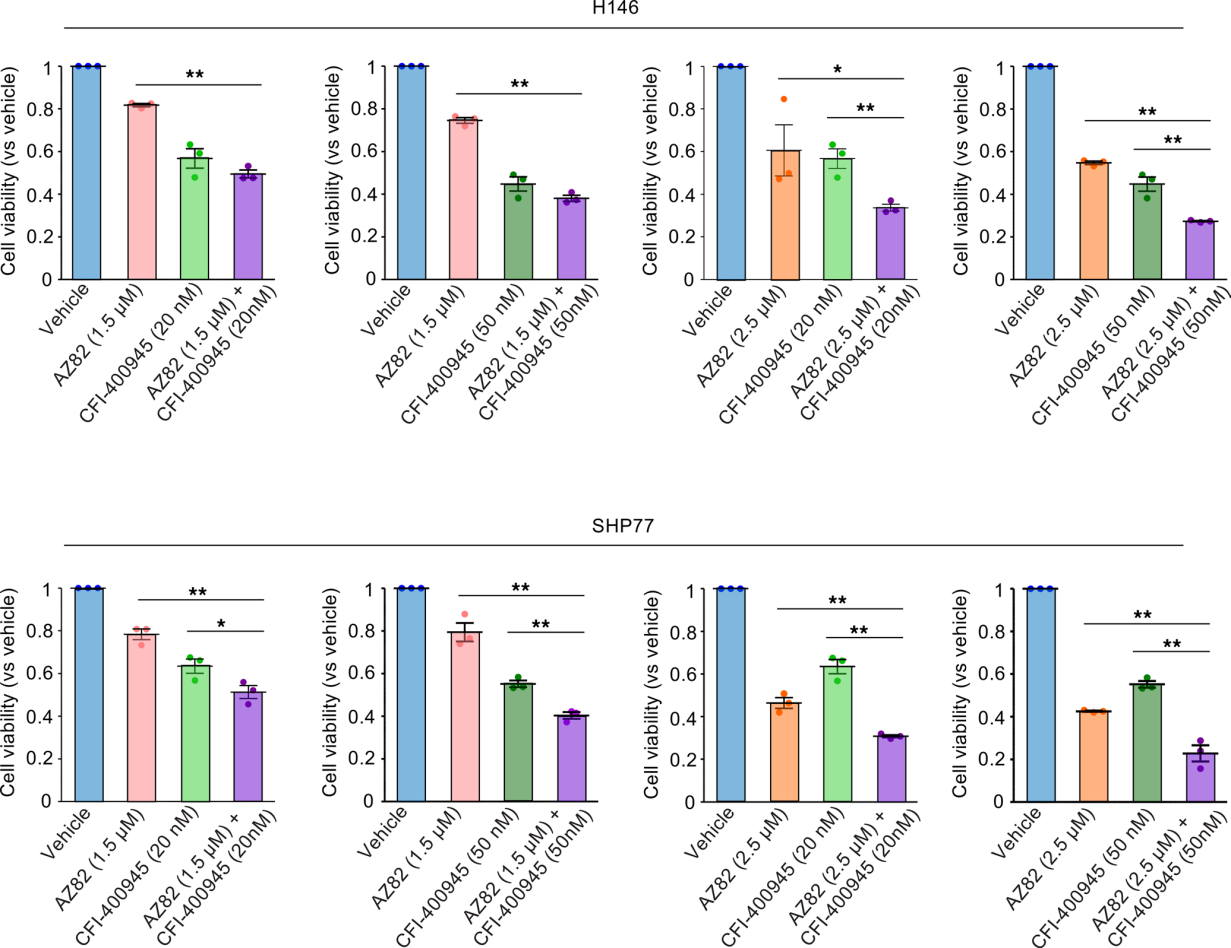
Cooperative growth-inhibitory effects of combined KIFC1 inhibition and PLK4 modulation in SCLC cells. Cell viability of SCLC cell lines treated with the KIFC1 inhibitor AZ82, the PLK4 modulator CFI-400945, or their combination at the indicated concentrations. Error bars represent standard deviation. *P* values were calculated by Dunnett’s test. *P < 0.05; **P < 0.01.

Because concurrent centrosome amplification and inhibition of centrosome clustering are expected to enhance the induction of multipolar cell division, we examined the combined effects of AZ82 and CFI-400945 using 2 concentrations of each drug. As anticipated, the combination treatment resulted in significantly greater growth inhibition than either monotherapy (Figure 10). Bliss independence analysis indicated predominantly additive interactions, with mild synergy observed at certain concentration pairs.

These results indicate that dual targeting of PLK4 and KIFC1 effectively enhances growth suppression through complementary mechanisms.

## Discussion

This study demonstrated that inhibition of KIFC1 exerts substantial antineoplastic effects in SCLC by triggering anaphase catastrophe. These effects result from the disruption of centrosome clustering, leading to multipolar mitosis, as observed in both relevant in vitro and in vivo models.

Previous studies linked KIFC1 expression to smoking history, genomic instability, and DNA damage (33, 45, 61), features that are characteristic of SCLC. Consistently, our in silico analyses and in vitro experiments revealed substantially elevated KIFC1 expression in SCLC compared with normal tissues and other cancer types (Figures 1 and 2). We recognize that these comparisons should be interpreted with caution, as the cell line analyses included only one nonmalignant and one NSCLC model, and because publicly available pan-cancer proteomic datasets including SCLC samples are lacking, likely owing to the rarity of surgical resections for this disease. To address this limitation, we directly assessed KIFC1 protein expression in clinical SCLC specimens using IHC across 2 independent TMAs as shown in Figures 3 and 4. This analysis demonstrated higher KIFC1 protein levels in SCLC tissues compared with normal lung and NSCLC samples, providing protein-level support for KIFC1 upregulation in SCLC. Although several biochemical functions of KIFC1 have been described, its role in the biology of differentiated normal cells remains unclear (33, 40, 41, 62, 63). Prior studies (15, 47–49), together with our own findings, indicate that KIFC1 inhibition exerts limited effects on certain noncancerous somatic cell types, including bronchial epithelial cells, under the experimental conditions tested. At the same time, recent reports have demonstrated that KIFC1 inhibition can perturb centrosome dynamics in specific normal cell contexts, such as germ cells, underscoring that the consequences of KIFC1 inhibition are cell type and context dependent (62, 64, 65). Taken together with its marked overexpression in SCLC, these complementary findings support the potential of KIFC1 as a therapeutic target with a context-dependent therapeutic window in SCLC.

KIFC1 is essential for the clustering of supernumerary centrosomes in cancer cells (37). SCLC is characterized by near-universal inactivation of p53, which is associated with centrosome amplification (55, 56). In this study, immunostaining for pericentrin (Figure 2, A and B) revealed that SCLC cells harbor supernumerary centrosomes more frequently than normal lung, NSCLCs, or other tumor cell types previously studied (60). Our in silico analysis further showed that PLK4, a master regulator of centrosome duplication, is overexpressed in SCLC, and positively correlates with KIFC1 expression (Figure 1, C–E). Prior work reported that KIFC1 stabilizes PLK4 by inhibiting the E3 ubiquitin ligase TRIM-37 (41). Together, these findings suggest a close functional interaction between KIFC1 and PLK4, supporting the view that KIFC1 plays a critical role in cells with supernumerary centrosomes and is upregulated in response to centrosome amplification in SCLC. Moreover, our IHC analyses of clinical specimens and scRNA-seq analyses demonstrated a positive correlation between KIFC1 and Ki67 expression (Figure 4, D–G), linking KIFC1 expression to highly proliferative activity in SCLC. Consistent with this proliferative phenotype, survival analysis in our TMA cohort showed a trend toward shorter overall survival with higher KIFC1 expression (*P* = 0.021, log-rank test for trend; Supplemental Figure 4). Collectively, these findings highlight the biological and potential clinical significance of KIFC1 in SCLC and underscore the rationale for targeting KIFC1 in SCLC.

Previous studies described in vitro effects of KIFC1 inhibition in several cancer types, including NSCLC and prostate cancer (38, 44–46). Here, we comprehensively investigated the effects of KIFC1 inhibition in SCLC both in vitro and in vivo, and demonstrated consistent antitumor activity, likely mediated through disruption of centrosome clustering. Notably, our immunostaining analyses revealed that the increase in multipolar mitosis observed after KIFC1 inhibition was accompanied by a corresponding decrease in pseudo-bipolar mitosis, while the total number of cells with supernumerary centrosomes remained unchanged (Figure 7, E and F, Figure 9G, and Supplemental Figures 7 and 8). These findings indicate that KIFC1 inhibition triggers multipolar mitosis and subsequent antineoplastic effects in SCLC by disrupting the clustering of preexisting supernumerary centrosomes, thereby triggering anaphase catastrophe (28–30). Anaphase catastrophe occurs predominantly in cancer cells with supernumerary centrosomes, whereas cells with 2 centrosomes are generally less susceptible. Consistent with this principle, under the experimental conditions tested, KIFC1 inhibition exerted minimal effects on proliferation and mitotic spindle organization in noncancerous bronchial epithelial cells (NHBE and BEAS-2B) in our study, as shown in Supplemental Figure 9. In addition to these cellular findings, our transcriptomic analysis revealed coordinated changes in genes involved in centrosome organization, spindle assembly, and cell cycle regulation following KIFC1 knockdown (Figure 8). Notably, while overlap at the individual-gene level between genes upregulated in KIFC1-high tumor cells (Supplemental Figure 5) and genes downregulated upon KIFC1 depletion was limited, both datasets converged at the pathway level: mitotic and centrosome-related programs enriched in KIFC1-high cells were broadly attenuated after KIFC1 knockdown, whereas checkpoint- and apoptosis-related pathways were relatively increased. Together, these transcriptional changes are consistent with mitotic stress induced by centrosome-clustering failure and support the mechanistic link between KIFC1 inhibition and tumor cell death in SCLC.

Using time-lapse fluorescence microscopy, we previously demonstrated that multipolar anaphase induced by CDK2 inhibition can lead to diverse cell fates besides apoptosis (66). In the present study, as shown in Figure 6, both cell cycle arrest and apoptosis were observed following KIFC1 inhibition in SCLC cells. Consistent with these observations, treatment with the pan-caspase inhibitor Q-VD-OPh partially alleviated AZ82-induced growth inhibition (Supplemental Figure 6), indicating that caspase-dependent apoptosis contributes to — but does not fully account for — the overall growth suppression. These findings suggest that a subset of cells may survive multipolar mitosis and subsequently arrest at the G_1_/S checkpoint as a result of mis-segregated chromosome. Notably, the magnitude of growth inhibition observed in CRISPR-depleted cells exceeded the extent of apoptosis detected at single time points. This likely reflects the cumulative and sustained impact of KIFC1 loss on mitotic fidelity and proliferative capacity, whereas the apoptosis assays capture only early events within the surviving population. While prior work using live-cell imaging has provided detailed mechanistic insight into these processes, our present study focused on establishing the functional and translational relevance of KIFC1 inhibition in SCLC. Future studies using live-cell imaging will help clarify the temporal dynamics and heterogeneity of these cellular responses. In addition, it should be noted that direct demonstration that cell death in vivo arises from multipolar division will require further mechanistic investigation, such as live or intravital imaging approaches.

A limitation of the therapeutic strategies involving anaphase catastrophe is that they theoretically spare not only normal cells but also cancer cells lacking supernumerary centrosomes. Although our study confirmed that supernumerary centrosomes are prevalent in SCLC cells, a subset lacked this feature (Figure 2B) and may therefore be less susceptible to anaphase catastrophe. Importantly, accumulating evidence indicates that cells with supernumerary centrosomes often constitute genomically unstable subpopulations that can disproportionately influence tumor growth and therapeutic resistance (14, 67–69). In this context, the preferential elimination of these KIFC1-dependent cells could plausibly reduce overall tumor aggressiveness, even if diploid-like cells remain viable. Future studies will be needed to determine how centrosomally normal populations adapt or compensate following KIFC1 inhibition in vivo.

In light of these considerations, we examined whether pharmacological modulation of PLK4 with CFI-400945 could cooperate with KIFC1 inhibition to intensify mitotic disruption in SCLC (Figure 10). In our previous study, CFI-400945–mediated alteration of PLK4 activity was shown to induce centrosome amplification and exacerbate mitotic defects, and these abnormalities became more detrimental when centrosome clustering was simultaneously impaired (60). Building on this mechanistic foundation, the present findings demonstrate that combining CFI-400945 with AZ82 enhances growth suppression beyond either agent alone (Figure 10). These results extend our earlier observation that PLK4 modulation and impaired centrosome clustering act cooperatively to heighten mitotic vulnerability (60), supporting cotargeting of centrosome amplification and clustering as a biologically rational therapeutic strategy for SCLC.

In conclusion, we demonstrated that SCLC is characterized by a high frequency of supernumerary centrosomes and elevated expression of KIFC1. Pharmacological and genetic inhibition of KIFC1 disrupted centrosome clustering, induced multipolar mitosis, and exerted potent antineoplastic effects in SCLC in vitro and in vivo. These findings reveal an underexplored vulnerability in SCLC and propose induction of anaphase catastrophe via KIFC1 inhibition as a promising therapeutic strategy. Given the lethality of SCLC and the lack of effective therapies, advances in SCLC therapeutics are needed. We acknowledge that AZ82, the KIFC1 inhibitor used in this study, may exhibit off-target cytotoxic effects in addition to its intended activity, and we have accordingly tempered our interpretation of its specificity. Nonetheless, several phenotypic similarities between AZ82 treatment and genetic KIFC1 depletion support that the antitumor effects observed are at least partly KIFC1-dependent. Future studies incorporating inducible KIFC1 knockdown systems and the development of more selective, drug-like KIFC1 inhibitors will be essential to clarify the in vivo contribution of KIFC1 inhibition and facilitate clinical translation.

## Methods

### Sex as a biological variable.

All animal experiments in this study were performed using male mice to minimize variability associated with hormonal cycles and to enhance reproducibility. Analyses of human tissue samples, which included both male and female donors, yielded comparable results, suggesting that the findings are relevant to both sexes.

### In silico analyses.

Lung cancer patient datasets (GSE30219, GSE43346, and GSE40275) were retrieved from the GEO database (http://www.ncbi.nlm.nih.gov/geo). The GSE30219 dataset includes 303 clinical specimens (21 SCLC, 268 NSCLC, and 14 normal lung tissues). The GSE43346 dataset includes 66 clinical specimens (23 SCLC and 43 normal tissues). The GSE40275 dataset includes 84 clinical specimens (26 SCLC, 15 NSCLC, and 43 normal lung tissues). Correlations between *PLK4* and *KIFC1* expression were assessed using cell line data from the CCLE (https://sites.broadinstitute.org/ccle/) and clinical samples from TCGA (https://portal.gdc.cancer.gov/). scRNA-seq data for SCLC were obtained from the GEO database (accession number GSE164404). Downstream analysis and visualization were performed using the Seurat package in R (version 5.1.0). Differential gene expression analysis between *KIFC1*-high and other SCLC clusters was performed using the FindMarkers function, with genes showing |log_2_ fold change| > 1 and adjusted *P* values < 0.05 considered as differentially expressed. Pathway enrichment of upregulated genes in *KIFC1*-high SCLC was analyzed using EnrichR (https://maayanlab.cloud/Enrichr/) with the top 200 differentially expressed genes.

### Cell culture.

The human NSCLC cell line A549, the SCLC cell lines H146, SHP77, H209, H524, and H526, and the immortalized bronchial epithelial cell line BEAS-2B were purchased from and authenticated by American Type Culture Collection. The NHBE cell line was purchased from Lonza. Cells were cultured at 37°C with 5% CO_2_ in a humidified incubator. NSCLC and SCLC cell lines, as well as BEAS-2B cells, were maintained in RPMI 1640 medium (Fujifilm Wako) with 10% fetal bovine serum, whereas NEBE cells were cultured in BEBM medium (Lonza).

### Immunocytochemistry for spindles and centrosomes.

Cells were fixed, stained with anti–α-tubulin and anti-pericentrin specific antibodies along with Hoechst, and then mounted with ProLong Gold antifade reagent (P36934, Invitrogen). Stained cells were scored for multipolar anaphase cells using the APEXVIEW APX100 fluorescence microscope (EVIDENT). At least 100 mitotic cells per sample were evaluated. Primary antibodies were α-tubulin (T6199, Sigma-Aldrich; 1:1,000) and pericentrin (ab4448, Abcam; 1:1,000). Secondary antibodies were Goat anti-Mouse IgG (H+L) Cross-Adsorbed Secondary Antibody, Alexa Fluor 594 (A11005, Invitrogen; 1:500), and Goat Anti-Rabbit IgG H&L (FITC) (ab97050, Abcam; 1:150). Hoechst 33342 (62249, Thermo Fisher Scientific; 1:10,000) stained for DNA.

### Immunoblot assays.

Equal amounts of protein from total cell lysate were size-fractionated by SDS-PAGE (e-PAGEL, ATTO Corp.) and transferred to nitrocellulose membranes (Immobilon-P, Merck Millipore). Membranes were blocked with 5% nonfat dry milk solution (190-12865, Fujifilm Wako) and incubated with the indicated antibodies followed by detection using the ECL Select Western Blotting Detection Reagent (RPN2235, Cytiva). Primary antibodies used were anti-KIFC1 (ab172620, Abcam; 1:15,000) and anti-GAPDH (2118, Cell Signaling; 1:7,500). The secondary antibody used was goat anti-rabbit IgG (sc-2030, Santa Cruz Biotechnology; 1:5,000).

### IHC.

Two sets of SCLC TMAs were analyzed by IHC. One was commercially available TMA (LC1402, TissueArray.Com LLC) with 70 paired SCLC specimens and adjacent normal lung tissues. The other TMA was generated in our institute. Formalin-fixed paraffin-embedded tumor specimens were obtained from patients who underwent surgical procedures and were diagnosed with lung cancer (47 SCLC and 47 NSCLC) at Kanazawa University Hospital between 2001 and 2021. Two cores were extracted from each specimen to create a TMA using tissue microarrayers. The study received approval from the institutional ethical committee of Kanazawa University (approval 12644-2). Sections were deparaffinized, rehydrated, and subjected to antigen retrieval using Antigen Unmasking Solution, citric acid based (H-3300, Vector), in a pressure cooker. Blocking of nonspecific binding was performed using ImmunoBlock (CTKN001, KAC) for 5 minutes at room temperature. The sections were incubated with the primary antibody, anti-KIFC1 antibody (HPA055997, Merck; 1:1,000), for 30 minutes at 37°C. Histofine Simple Stain MAX PO (414341, Nichirei Biosciences) was used as the secondary antibody, and the reaction was carried out at 37°C for 30 minutes. To visualize the antigen-antibody complex, an ImmPACT DAB substrate kit (Vector Laboratories) was used. KIFC1 expression of commercial TMA (LC1402) was semiquantitatively evaluated by a board-certified pathologist based on the percentage of positively stained tumor cells. The scoring system was defined as follows: score 0, <1% positive cells; score 1, 1%–10%; score 2, 10%–30%; and score 3, >30%. KIFC1 expression of our in-house TMA was assessed quantitatively independently by 2 pathologists based on the percentage of positively stained tumor cells.

### KIFC1 inhibition in vitro.

AZ82, a specific KIFC1 inhibitor (70, 71), was purchased from MedChemExpress. Two kinds of siRNAs targeting KIFC1 (s7906, s7907) and a scrambled siRNA (Negative Control No. 1) were purchased from Thermo Fisher Scientific. Transient transfection was achieved using Lipofectamine RNAiMAX (Thermo Fisher Scientific) according to the manufacturer’s protocols. For CRISPR/Cas9–mediated depletion of KIFC1, sgRNA targeting KIFC1 or control sgRNA was delivered via lentivirus, as previously described (72). The designed sgRNA sequence for targeting KIFC1 was TCCCGGTCTGACGAGCGGCGTGG. The sgRNA sequence for negative control was GCACTACCAGAGCTAACTCA. Knockout was confirmed by immunoblot assays.

### RNA isolation and RT-qPCR assays.

Total RNA from cultured cells was extracted using RNAiso Plus (Takara Bio). The purity and concentration of RNA were determined by NanoDrop ONE (Thermo Fisher Scientific). Subsequently, RNA was reverse-transcribed into cDNA using SuperScript III Reverse Transcriptase (Thermo Fisher Scientific). RT-qPCR was performed with THUNDERBIRD SYBR qPCR Mix (Toyobo) on a 7500 Fast Real-Time PCR System (Applied Biosystems) according to the manufacturer’s protocols. Gene expression was normalized to *GAPDH*, and the relative mRNA expression of target genes was calculated using the ΔΔCt method. Specific primers for *KIFC1* and *GAPDH* were designed via NCBI Primers BLAST (https://www.ncbi.nlm.nih.gov/tools/primer-blast/), as follows: *KIFC1* forward, 5′-AGAACTTGCGTGCTTGTGTC-3′; *KIFC1* reverse, 5′-TTCTTCCCGCTCAGTCAGTAAG-3′; *GAPDH* forward, 5′-CACCACCAACTGCTTAGCAC-3′; *GAPDH* reverse, 5′-TGGCAGGTTTTTCTAGACGG-3′.

### Cell proliferation assays.

Logarithmically growing cells were seeded at optimized densities for each cell line into 12- or 24-well tissue culture plates. To test the effects of pharmacological inhibition of KIFC1, cells were treated with AZ82 or vehicle (DMSO) for 96 hours, and then cell viability was assessed in each concentration compared with vehicle. To test the effects of genetic inhibition of KIFC1, cell proliferation was tracked for 4–7 days and compared with day 0. Proliferation was measured using the CellTiter-Glo Luminescent Assay (Promega). Luminescence was measured with ARVO X5 (PerkinElmer). For caspase inhibition experiments, cells were treated with AZ82 (2.5 μM) in the presence or absence of the pan-caspase inhibitor Q-VD-OPh (5–10 μM; MedChemExpress) for 48 hours, and cell viability was determined using the same assay. For drug combination studies with the specific PLK4 modulator CFI-400945 (MedChemExpress), cells were cotreated simultaneously with AZ82 and CFI-400945 at 2 concentrations of each compound for 72 hours, and cell viability was determined using the same assay.

### Apoptosis assays.

Logarithmically growing cells were seeded at optimized densities for each examined cell line into individual wells of 6-well tissue culture plates. Apoptosis assays were performed with annexin V/propidium iodide staining using the FITC Annexin V and Apoptosis Detection KIT I (BD Pharmingen) after 48 hours of KIFC1 inhibition. Stains were measured using a Gallios flow cytometer (Beckman Coulter) and then analyzed with Kaluza software (Beckman Coulter).

### Cell cycle analyses.

Cells were fixed in ice-cold 70% ethanol overnight, incubated with RNase (R4875, Sigma-Aldrich) at 37°C for 20 minutes, and then stained with propidium iodide (169-26281, Fujifilm Wako) at 4°C for 15 minutes. DNA contents were measured using a Gallios flow cytometer after 24–48 hours of exposure to AZ82 or 72–96 hours after KIFC1 knockdown using siRNAs. For synchronization, cells were exposed to 1 μg/mL of aphidicolin for 24 hours before AZ82 treatment or KIFC1 knockdown as previously described (73, 74).

### RNA sequencing and transcriptomic analyses.

Logarithmically growing H146 cells were seeded at optimized densities into individual wells of 6-well tissue culture plates. Total RNA was extracted 48 hours after siRNA transfection and submitted to Rhelixa Inc. for library preparation and RNA sequencing. Raw RNA sequencing reads in FASTQ format were aligned to the reference genome (GRCh38) using HISAT2 (version 2.2.1) (https://github.com/DaehwanKimLab/hisat2) with default parameters. Gene-level expression quantification was performed using featureCounts (version 2.0.8). Differential expression analysis between the 2 groups was conducted using DESeq2 (version 1.46.0) in R (version 4.4.2).

### In vivo experiments.

All animal studies and procedures were approved by the Ethics Committee of The University of Tokyo (approval A2023M089). Mice were maintained under specific pathogen–free conditions with a 12-hour light/12-hour dark cycle for 7 continuous days before use. H146 or SHP77 cells (6 × 10^6^) were mixed with Matrigel (Corning) and injected subcutaneously into 6-week-old male nude mice (BALB/cSlc-nu/nu, Japan SLC). Mice with palpable tumors were treated with 12.5 μg/g of AZ82 or vehicle (DMSO) 4 times weekly for 3 weeks by intraperitoneal injection (*n* = 7 per group). Body weights and tumor volumes were measured twice a week. Tumor volume (V) was calculated as V = (length × width^2^)/2. After treatment, tumors were resected from sacrificed mice and weighed. Resected tumors were fixed with 10% formalin for 24 hours and were paraffin-embedded. For mitosis assessment, sections were probed with anti-pericentrin antibody (ab220784, Abcam; 1:1,000) and counterstained with hematoxylin. Stained cells were scored for multipolar mitotic cells using APEXVIEW APX100. Additionally, TUNEL assays were performed to evaluate apoptosis in resected tumors.

### Statistics.

Differences between analyzed groups were assessed by a Student’s *t* or Mann-Whitney *U* test. To control the overall type I error rate in addressing the multiple comparisons, Tukey’s method was used for all pairwise comparisons across cell lines or experimental conditions. Dunnett’s method was applied for comparing the result of different drug concentrations with the control (vehicle) group, as well as comparing KIFC1-targeting siRNA effects with control siRNA. Kaplan-Meier survivals were calculated by the log-rank test. Statistical analyses were conducted with R (version 4.4.2) and GraphPad Prism software (version 6, GraphPad Software). All statistical tests were 2-sided, and *P* values less than 0.05 were considered significant.

### Study approval.

IHC studies using our in-house TMA were approved by the institutional ethical committee of Kanazawa University (approval 12644-2). All animal studies and procedures were approved by the Ethics Committee of The University of Tokyo (approval A2023M089).

### Data availability.

RNA sequencing data were deposited in the GEO database under accession number GSE318538. Supporting data values for all figures are provided in the Supporting Data Values file. Additional raw data are available upon reasonable request. No custom analytic code was generated for this study.

## Author contributions

M Kawakami conceived and designed the study. NN, M Toda, AK, MH, M Tokunaga, HI, M Ka, TI, TA, KW, YO, and M Kawakami developed methodology. NN, M Toda, MH, M Tokunaga, MS, YI, YO, and M Kawakami acquired data. NN, M Toda, MH, HI, YO, XL, ED, HK, and M Kawakami analyzed and interpreted data. NN, ED, and M Kawakami wrote the manuscript. MH, YO, XL, ED, and M Kawakami provided administrative, technical, or material support. M Kawakami supervised the study.

## Funding support

This work is the result of NIH funding, in whole or in part, and is subject to the NIH Public Access Policy. Through acceptance of this federal funding, the NIH has been given a right to make the work publicly available in PubMed Central.

- Princess Takamatsu Cancer Research Fund (to M Kawakami).
- Takeda Science Foundation (to M Kawakami).
- Japan Society for the Promotion of Science KAKENHI Grants 21H02776 and 24K02316 (to M Kawakami).
- Partial support by the National Cancer Institute and National Institutes of Health contract 75N91019D00024 (to XL and ED).
- Partial support by the Frederick National Laboratory’s Laboratory Directed Exploratory Research program (to XL).

## Supplementary Material

Supplemental data

Unedited blot and gel images

Supporting data values

## Version 1 04/08/2026

Electronic publication
