# Supplementary material for: Targeting KIFC1 to disrupt centrosome clustering and trigger anaphase catastrophe in small-cell lung cancer
Source: JCI Insight. 2026 Apr 8;11(7):e199352. doi: 10.1172/jci.insight.199352 (PMC13134724; doi:10.1172/jci.insight.199352)
Supplement: Supplemental data [file jciinsight-11-199352-s116.pdf]

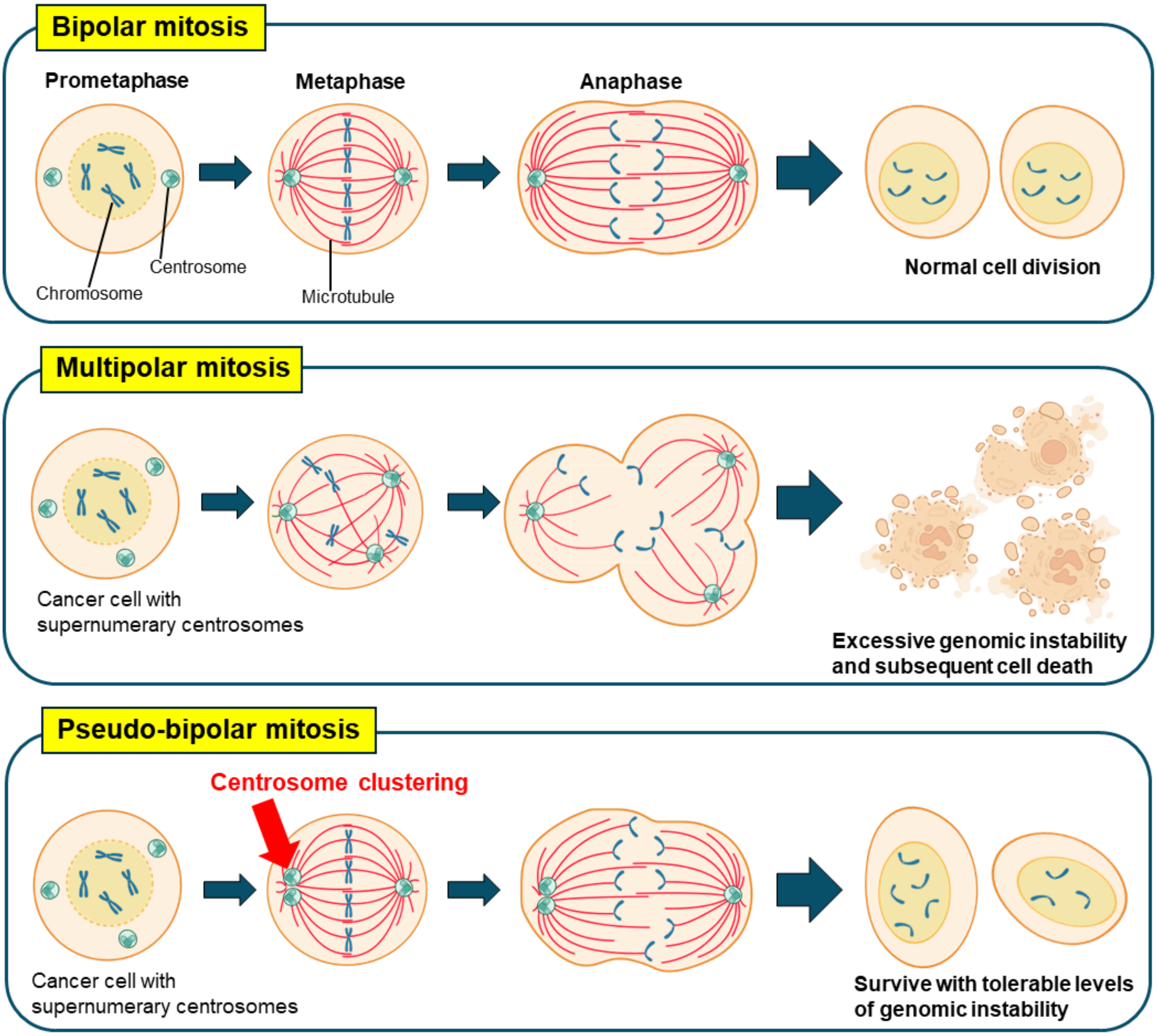

Supplemental Figure 1. Three types of cell mitosis.

Normal cells with two centrosomes form bipolar spindles and undergo bipolar mitosis dividing into two daughter cells (upper panels). On the other hand, cancer cells with supernumerary centrosomes undergo multipolar mitosis that may lead to subsequent cell death due to excessive chromosome mis-segregation (middle panels). To avoid this detriment process, cancer cells cluster their supernumerary centrosomes into two poles, which enables cells to form bipolar spindles and undergo mitosis similar with normal cells (pseudo-bipolar mitosis) that leads to tolerable levels of genomic instability (lower panels).

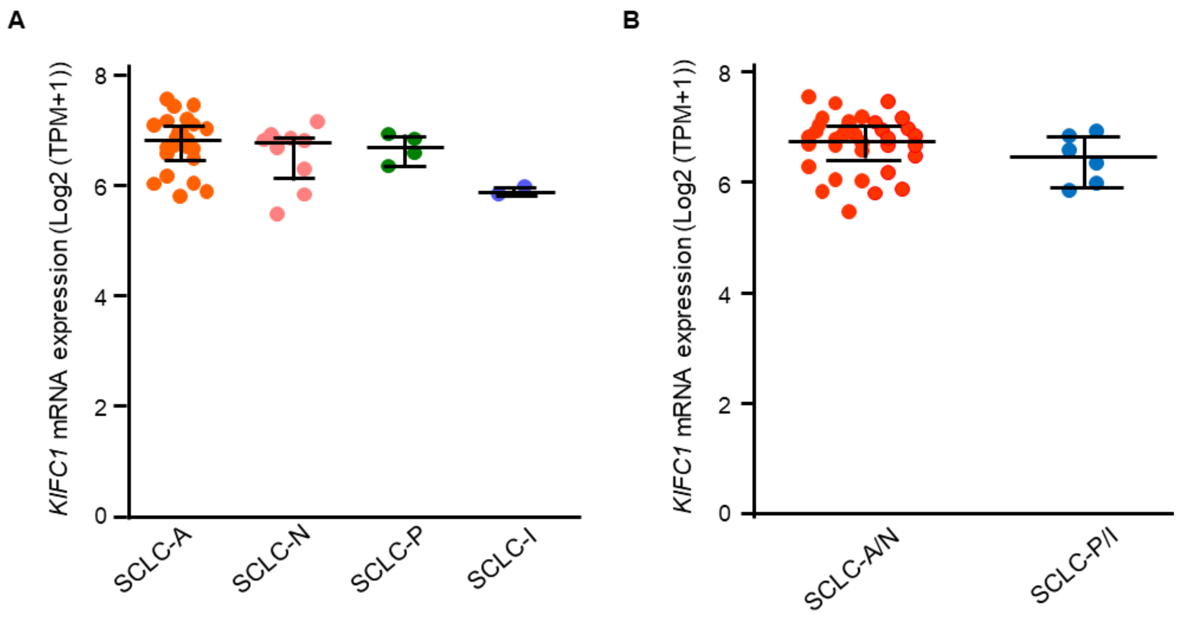

Supplemental Figure 2. Comparison of *KIFC1* mRNA expression among SCLC transcriptional subtypes in the CCLE dataset.

(A) *KIFC1* mRNA expression among SCLC-A, SCLC-N, SCLC-P, and SCLC-I subtypes. **(B)** Combined analysis of SCLC-A/N subtypes (SCLC-A, SCLC-A+N, and SCLC-N) versus SCLC-P/I subtypes (SCLC-P and SCLC-I) for reference. Each dot represents a single sample. The *p* values were obtained by using a two-sided *t* test with multiple comparisons adjusted using Tukey's method. No significant differences were observed among subtypes. Bars represent median values and interquartile range.

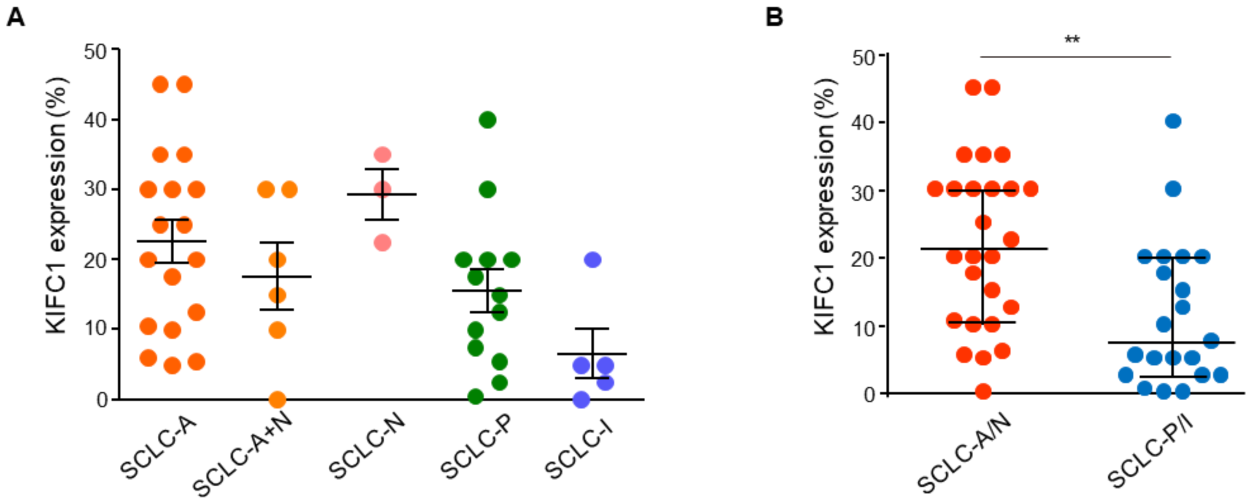

Supplemental Figure 3. Immunohistochemical comparison of KIFC1 protein expression among SCLC transcriptional subtypes.

(A) KIFC1 protein expression among SCLC-A, SCLC-N, SCLC-P, and SCLC-I subtypes. **(B)** Combined analysis of SCLC-A/N subtypes (SCLC-A, SCLC-A+N, and SCLC-N) versus SCLC-P/I subtypes (SCLC-P and SCLC-I) for reference. KIFC1 expression was higher in the SCLC-A/N subgroup. Each dot represents a single case. The p values were obtained by using a two-sided t test with multiple comparisons adjusted using Tukey's method. Double asterisk is $p < 0.01$. Bars represent median values and interquartile range.

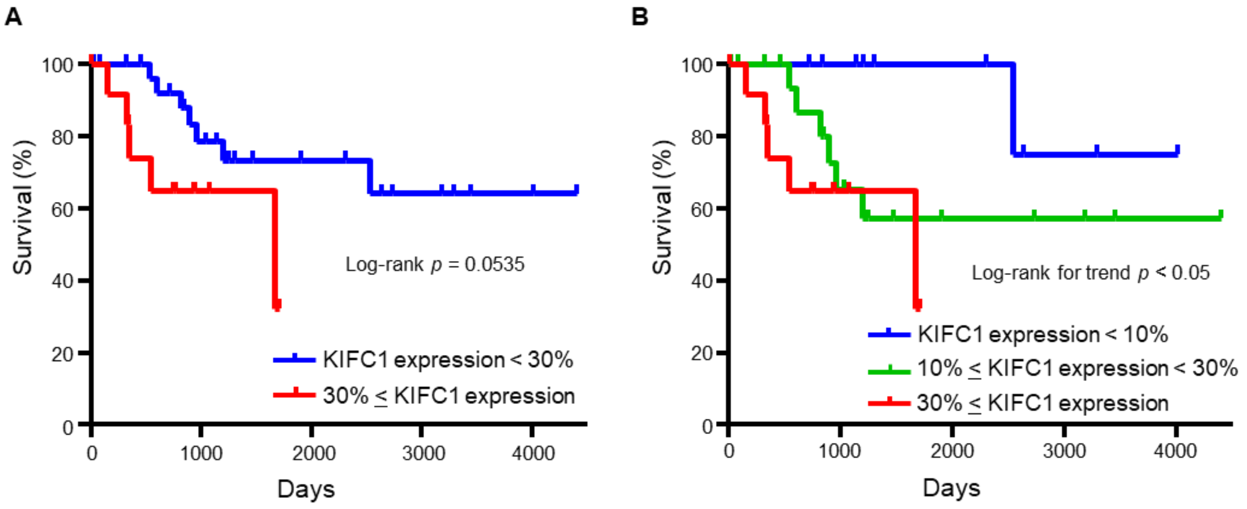

Supplemental Figure 4. Prognostic impact of KIFC1 expression in SCLC.

(A) Kaplan–Meier curve comparing patients dichotomized into KIFC1-high and KIFC1-low groups. A strong trend toward shorter overall survival was noted in the high-expression group (p = 0.053, log-rank test). **(B)** Kaplan–Meier analysis of overall survival in patients from the institutional SCLC TMA cohort stratified into three groups according to KIFC1 IHC expression levels (low, intermediate, and high). A trend toward poorer survival with increasing KIFC1 expression was observed (log-rank test for trend, $p < 0.05$).

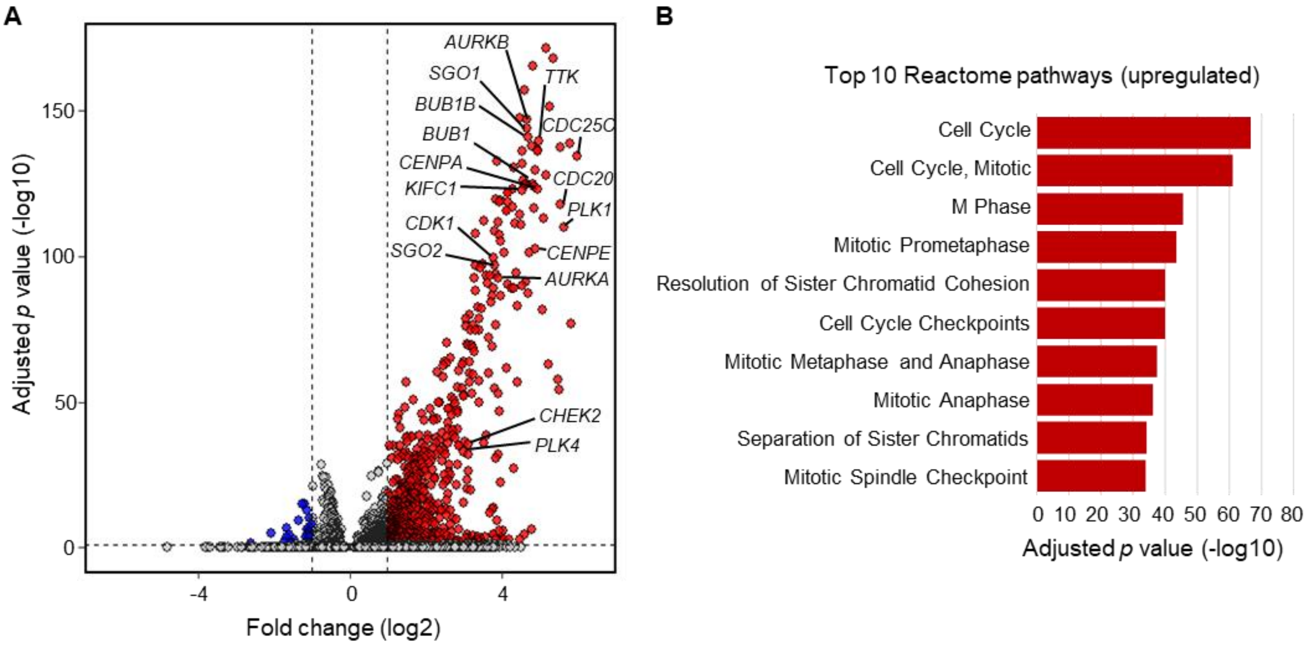

Supplemental Figure 5. Transcriptomic profiles of SCLC cells with high *KIFC1* expression.

(A) Volcano plots of differentially expressed genes between *KIFC1*-high SCLC cells and the remaining tumor cell population. Thresholds were set at $p < 0.05$ and absolute fold change (log2) > 1. Blue and red dots indicate downregulated and upregulated genes meeting these thresholds, respectively. **(B)** Top 10 upregulated pathways in *KIFC1*-high SCLC cells identified by GO analysis.

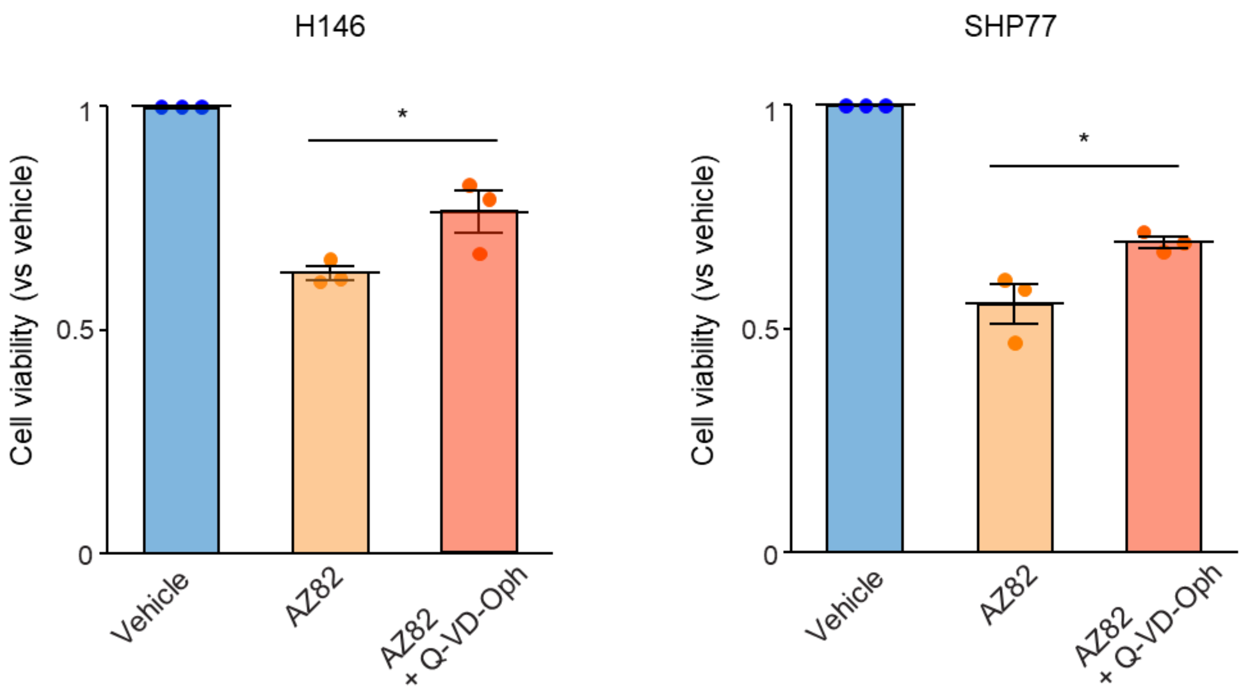

Supplemental Figure 6. Partial rescue of AZ82-induced viability loss by pan-caspase inhibition.

Cell viability (normalized to vehicle) in H146 and SHP77 treated with AZ82 (2.5 µM) ± Q-VD-OPh for 48 h. Error bars represent standard deviation. The *p* values were obtained by using a two-sided *t* test with multiple comparisons adjusted using Dunnett's method. Single asterisk is $p < 0.05$.

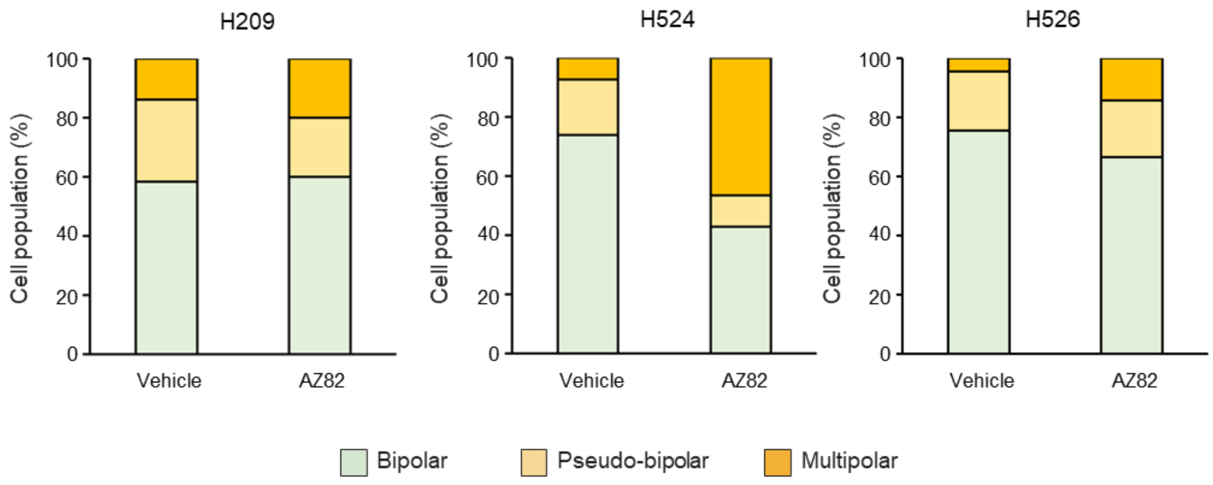

Supplemental Figure 7. Induction of anaphase catastrophe in SCLC cells following KIFC1 inhibition.

Distribution of bipolar, pseudo-bipolar, and multipolar SCLC cells after AZ82 treatment in H209, H524, and H526 cells.

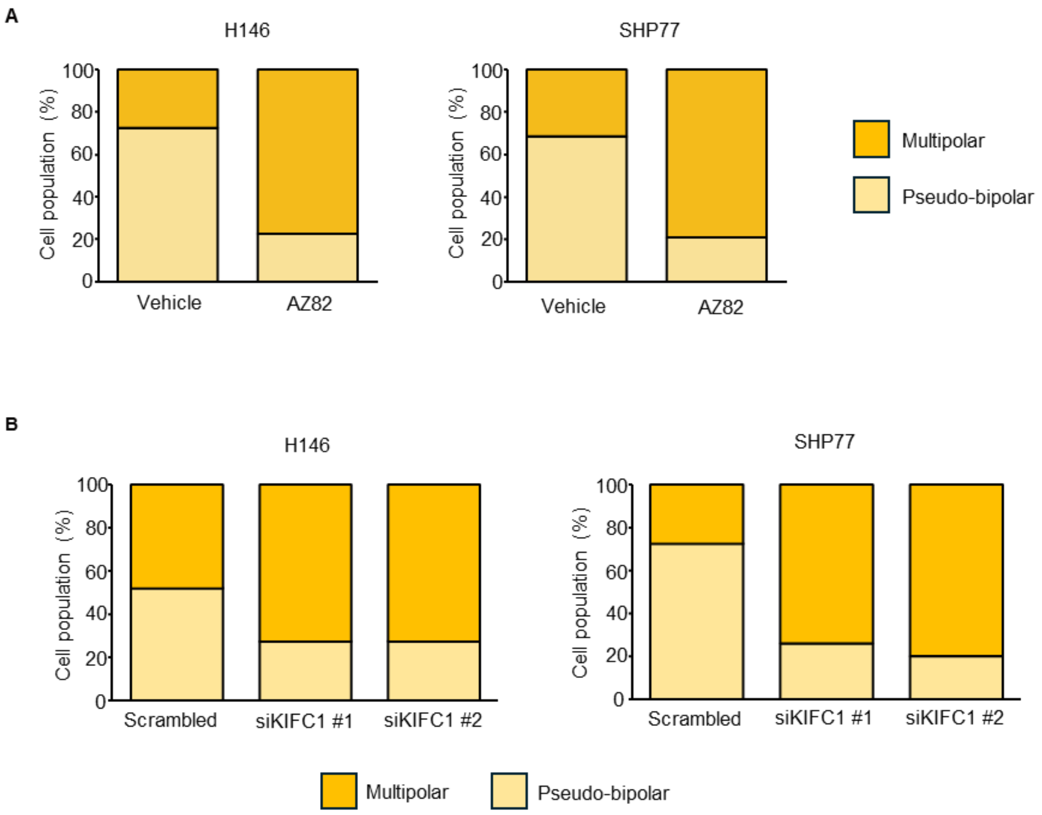

Supplemental Figure 8. Anaphase catastrophe induction in SCLC cells after KIFC1 inhibition.

Percentages of pseudo-bipolar and multipolar cell populations among SCLC cells with supernumerary centrosomes after independent KIFC1 inhibition by AZ82 **(A)** and siRNAs **(B)** are displayed. These data are derived from the same experiments as those summarized in Figure 7, E and F, and are presented here in an alternative format to separately visualize the pseudo-bipolar and multipolar subpopulations.

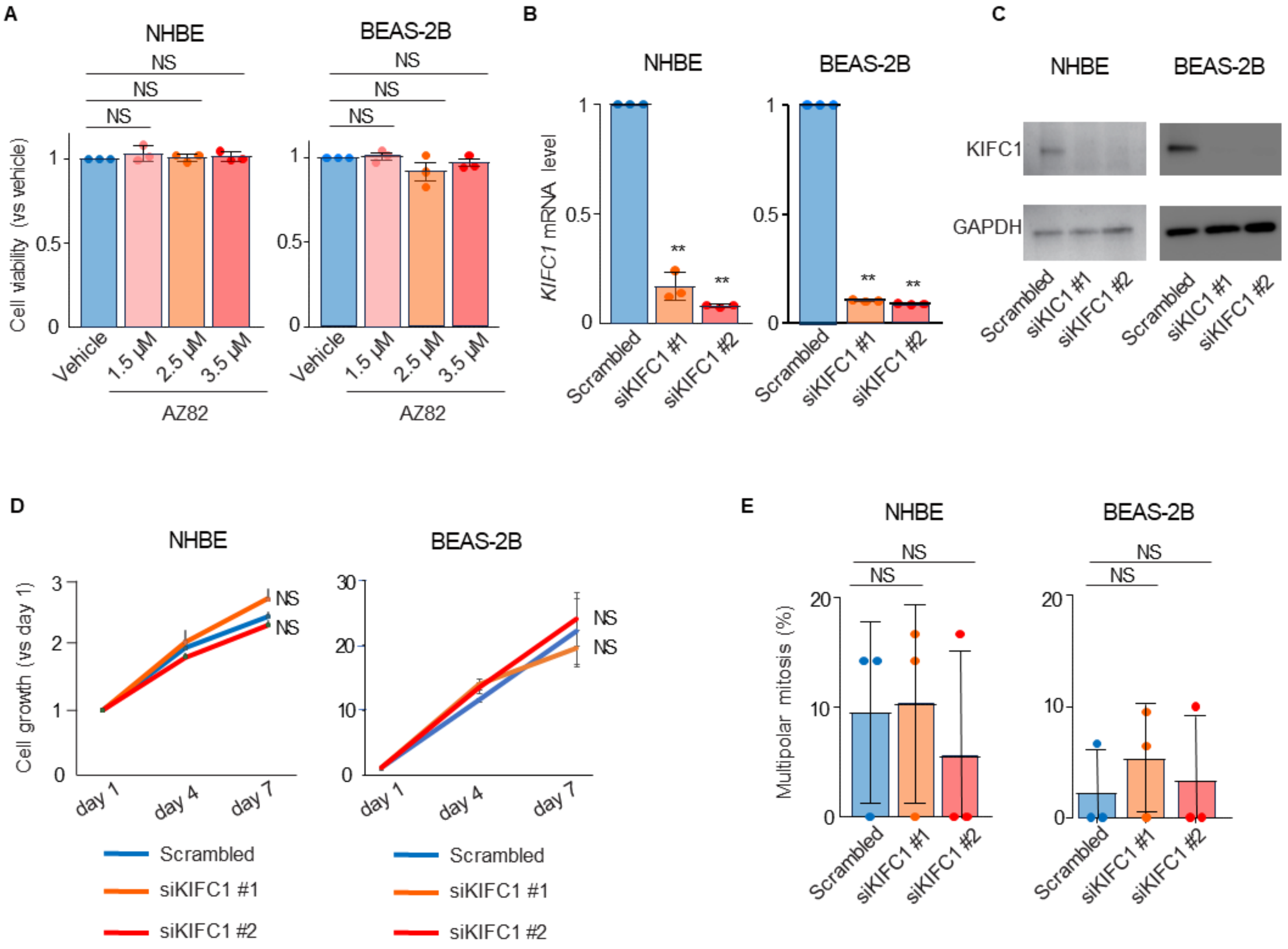

Supplemental Figure 9. KIFC1 inhibition in NHBE and BEAS-2B cells.

(A) AZ82 effects on cell viability of NHBE and BEAS-2B cells. **(B and C)** Knockdown of KIFC1 expression using siRNAs was confirmed by RT-qPCR **(B)** and immunoblot analysis **(C)**. **(D)** Cell proliferation curves of NHBE and BEAS-2B cells after KIFC1 knockdown. **(E)** Percentages of NHBE and BEAS-2B cells undergoing multipolar anaphase after KIFC1 knockdown.

Error bars are standard deviation. The *p* values were obtained by using a two-sided *t* test with multiple comparisons adjusted Dunnett's method. Double asterisk is $p < 0.01$. NS represents no significant difference.

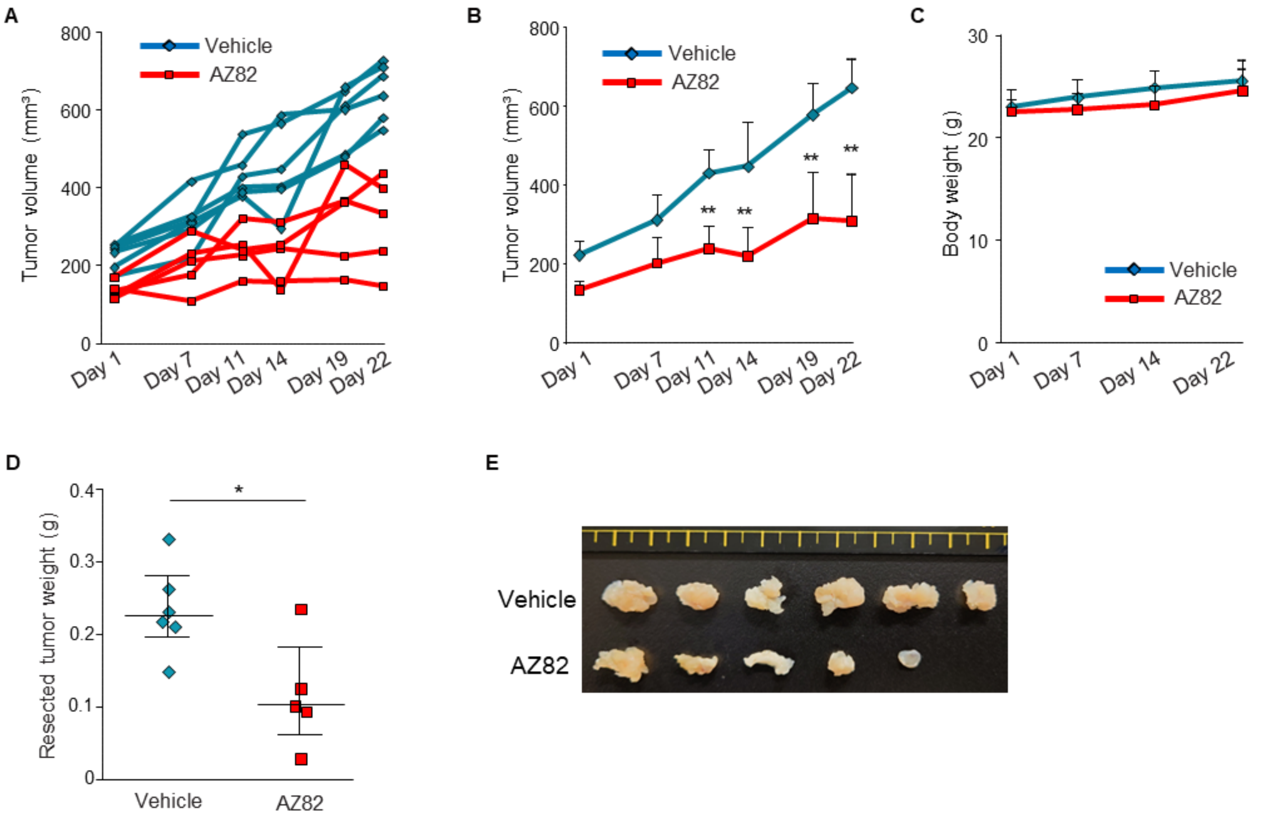

Supplemental Figure 10. In vivo antitumor effects of AZ82 treatment in SCLC.
(A and B) Comparison of tumor growth in SHP77-derived xenograft model treated with vehicle or AZ82. Spider plots showing tumor volume for each mouse are shown in **(A)**, and the average tumor volumes of the vehicle-treated and the AZ82-treated group over time are shown in **(B)**. Error bars represent standard deviation. *p* values were calculated using a two-sided *t* test. Double asterisk is $p < 0.01$. **(C)** Body weights of mice did not appreciably change during treatment with vehicle or AZ82. **(D)** Comparison of resected tumor weights after treatment with vehicle or AZ82. Each dot represents a single mouse. Bars indicate median values and interquartile range. *p* values were calculated using two-sided *t* tests. Single asterisk is $p < 0.05$. **(E)** Photographs of resected tumors from the vehicle-treated and the AZ82-treated groups.

Supplemental Table 1. SCLC patient characteristics of our in-house TMA.

Characteristics	Patient (n=47)
Age, median (IQR)	72 (66.5, 77)
Sex, Male, n (%)	43 (91)
Smoking history	
Current, n (%)	11 (23)
Former, n (%)	31 (66)
Never, n (%)	4 (9)
ND, n (%)	1 (2)
Stage	
IA, n (%)	11 (23)
IB, n (%)	13 (28)
IIA, n (%)	4 (9)
IIB, n (%)	5 (11)
IIIA, n (%)	8 (17)
IIIB, n (%)	3 (6)
IVA, n (%)	2 (4)
ND	1 (2)
Molecular subtype	
NE type, n (%)	28 (60)
non-NE type, n (%)	19 (40)
TTF-1	
positive, n (%)	22 (47)
negative, n (%)	25 (53)

Supplemental Table 2. Significantly downregulated (A) or upregulated (B) genes in *KIFC1*-high cells.

A

Gene	log2FoldChange	*p* value
BAIAP3	-2.594607	0.0187645
LY6H	-2.089371	6.85E-06
SCG5	-1.717057	0.0158855
AVP	-1.674314	0.0001362
NTHL1	-1.650727	2.16E-07
ABHD14A	-1.60286	3.59E-05
EPHX1	-1.534174	0.0421138
MCM6	-1.480237	0.0265517
NME3	-1.471428	0.0024301
CHGA	-1.355789	6.25E-10
NLRP1	-1.24073	7.33E-16
C4orf48	-1.186215	6.05E-16
EXOSC5	-1.155795	0.0001473
PRELID1	-1.135747	1.27E-13
SNHG19	-1.121906	5.66E-07
REX1BD	-1.120706	3.59E-05
AC245595.1	-1.1177	0.0177301
RAB3B	-1.101574	4.39E-05
PWAR6	-1.095661	0.0217953
TRAPPC6A	-1.094267	4.73E-06
PMEPA1	-1.074425	0.0002812
CD63	-1.055742	9.47E-11
C19orf70	-1.05343	9.24E-08
PLPPR3	-1.048012	0.0472424
MIAT	-1.036593	0.0001422
NUDT14	-1.027246	1.45E-05
DLL3	-1.012524	1.08E-08
APRT	-1.002824	0.0073313

B

Gene	log2FoldChange	*p* value
CDC25C	6.0135209	5.68E-135
TICRR	5.8438246	1.54E-77
ASPM	5.8146563	1.72E-139
PLK1	5.6417724	1.42E-110
KIF18B	5.5659959	4.94E-138
CDC20	5.5454584	1.43E-118
PIF1	5.5300862	5.89E-55
FAM72C	5.4865461	2.74E-58
KIF23	5.3820797	1.46E-168
KIF2C	5.2611635	3.69E-152
FAM72D	5.2538254	1.08E-63
DEPDC1	5.1808661	2.19E-128
DLGAP5	5.179129	2.86E-172
KIF20A	5.1253865	6.19E-114
CCNB1	5.0767116	1.53E-82
TTK	4.9780717	2.73E-140
CENPA	4.9705842	5.82E-124
KIF4A	4.9596312	3.23E-137
MKI67	4.9469658	4.37E-137
CENPE	4.9056971	4.00E-103
CDCA2	4.9012734	2.75E-130
UBE2C	4.8745855	1.11E-124
CCNB2	4.8513763	1.81E-117
KIF14	4.8472084	1.41E-125
HJURP	4.8421126	5.01E-166
TROAP	4.8005977	1.38E-138
Z94721.1	4.7945392	5.53E-07
CDCA8	4.7484046	5.69E-102
BUB1B	4.7100969	7.64E-142
IQGAP3	4.7049406	4.99E-88
SGO1	4.6876566	8.47E-145
AURKB	4.6822396	1.33E-147
HMMR	4.6543886	1.49E-125
CCNA2	4.636951	4.22E-92
CKAP2L	4.6203896	8.49E-158
CEP55	4.6148819	3.11E-125
RBM15-AS1	4.5977042	3.43E-05
BUB1	4.5672535	6.71E-127
NUF2	4.5646237	1.10E-132
NDC80	4.5594203	6.66E-137
NCAPH	4.5546779	1.40E-123
CENPF	4.5445352	1.00E-90
CIT	4.5254283	1.32E-111
C9orf84	4.5004415	0.0011601
PBK	4.4840205	3.26E-148
SKA1	4.4762563	2.66E-115
FAM72B	4.4302676	2.20E-57
ERCC6L	4.4164078	7.74E-84
NMU	4.3844221	5.29E-95
TPX2	4.3453649	5.37E-112
CCNA1	4.3355101	4.48E-28
TOP2A	4.3311347	8.65E-90
ANLN	4.3170857	3.56E-131
NEK2	4.3128884	6.46E-124
GTSE1	4.2823422	1.04E-117
POLQ	4.2737761	5.61E-90
AC011498.3	4.2052176	0.0011601
ARHGAP11A	4.1846709	1.28E-119
KNL1	4.1839246	2.34E-122
KIF15	4.1794987	1.11E-122
GAS2L3	4.1730118	3.98E-91
KIF11	4.1360246	2.02E-116
ESCO2	4.1330323	1.73E-116
NEIL3	4.1266872	1.47E-62
AC084757.2	4.0826167	0.0011601
PATE1	4.0806682	0.0011601
DIAPH3	4.0743859	7.57E-102
AC245100.7	4.0453006	0.0011601
FCRLA	4.0303563	8.31E-07
SIGLEC10	4.0105879	0.0007318
KIFC1	3.9962101	5.54E-120
SHCBP1	3.9930846	2.83E-87
PRR11	3.9835708	5.98E-106
HASPIN	3.9781213	3.05E-23
NCAPG	3.9557468	6.52E-108
CDCA3	3.9537231	2.40E-119

AC007240.1	3.9520395	2.04E-47	*TREM1*	3.4384934	0.0007702
AC008750.7	3.9464652	8.31E-07	*DSC3*	3.4229939	0.0134709
CDKN3	3.9279398	1.59E-112	*E2F8*	3.4207781	6.13E-58
BORA	3.9265016	6.67E-33	*CIP2A*	3.4193855	1.68E-75
ARHGEF39	3.9220973	1.82E-53	*EME1*	3.4080127	1.72E-50
ECT2	3.9165835	3.28E-93	*KIF20B*	3.4012718	2.12E-79
BIRC5	3.8986805	2.67E-133	*NCAPD2*	3.3892025	3.78E-83
HIST1H2BM	3.8914251	4.67E-10	*VIP*	3.3805545	0.0014387
HCK	3.8695305	0.0014058	*UXT-AS1*	3.3754953	0.0002904
AURKA	3.8524424	3.00E-77	*SPC24*	3.3325869	1.25E-97
HIST1H3G	3.8483667	7.86E-95	*TACC3*	3.322811	9.24E-89
HIST2H2AB	3.848236	2.84E-31	*SPC25*	3.3132572	1.22E-108
PIMREG	3.8431142	4.05E-120	*NUSAP1*	3.3132244	2.95E-75
HIST1H2AJ	3.8310524	2.30E-55	*AC008121.2*	3.300306	0.0253564
SGO2	3.8228691	1.23E-97	*MAD2L1*	3.2916572	2.82E-93
SPAG5	3.8096483	1.42E-109	*MYBL1*	3.2905283	7.95E-68
PARPBP	3.8080126	5.09E-90	*KIF18A*	3.2698109	3.64E-60
PATE2	3.8054741	2.46E-14	*LCE1E*	3.2633805	9.37E-05
KCNJ3	3.8021885	3.51E-05	*SKA3*	3.260812	2.60E-77
CDK1	3.7990767	3.01E-100	*HIST1H2AL*	3.2495562	1.46E-69
MYLK2	3.7912276	2.16E-05	*HYLS1*	3.2399234	1.34E-53
AC012073.1	3.7668306	4.87E-14	*RRM2*	3.2378055	2.33E-70
RTKN2	3.756784	5.30E-87	*AC100802.1*	3.1922791	2.02E-20
KNSTRN	3.7496555	9.70E-70	*CKAP2*	3.1878706	1.69E-75
MXD3	3.7425797	4.04E-85	*FAM111A*	3.1763874	1.15E-64
POC1A	3.7058985	4.47E-94	*AC108673.2*	3.1754952	0.0258539
AL023803.2	3.6904831	1.42E-12	*INCENP*	3.1706498	1.81E-60
PSRC1	3.6658151	1.08E-60	*MND1*	3.1614471	9.67E-58
FBXO43	3.6551181	6.40E-73	*MELK*	3.1579375	7.21E-81
OIP5	3.6268758	1.24E-91	*ACRV1*	3.1465343	0.0087148
ARHGAP11B	3.6174692	2.20E-39	*DDIAS*	3.1465095	4.17E-36
DEPDC1B	3.6127401	3.34E-94	*HIST1H2BB*	3.1451191	4.51E-27
AC091057.4	3.5658457	0.000285	*STIL*	3.1438842	2.03E-70
HIST1H3B	3.5390257	8.39E-113	*AC015849.5*	3.1395324	0.0006871
REEP4	3.5377251	1.86E-36	*PTPRE*	3.1252576	1.22E-32
AC141424.1	3.5296759	0.0253564	*CKS1B*	3.0933092	9.85E-71
RACGAP1	3.5117377	2.61E-98	*C2orf48*	3.0922766	3.28E-24
HIST1H1B	3.4833285	5.25E-83	*AC024592.2*	3.0874098	4.34E-05
FOXM1	3.4480928	9.74E-97	*NOSTRIN*	3.0844295	8.69E-21

PRC1	3.0676117	2.78E-79	*CDKN2D*	2.6579726	5.49E-66
FAM83A	3.0625196	0.0008083	*FGF21*	2.6517563	5.48E-09
CENPW	3.0535448	1.01E-76	*FAM72A*	2.6476276	1.85E-13
HIST1H4C	3.0433131	4.38E-37	*PRSS3*	2.6433211	1.19E-25
HIST1H3C	3.0195939	7.82E-34	*SMTN*	2.6322856	1.08E-26
EXO1	3.0162664	1.16E-52	*DBF4*	2.6322436	1.69E-48
AL133215.2	3.0160102	2.34E-16	*HIST2H2AC*	2.6117766	2.32E-29
CENPI	3.013487	4.23E-53	*KIF24*	2.5989592	1.96E-43
WDR62	3.0061805	9.46E-65	*LINC02261*	2.5962784	4.21E-19
SMC4	2.9835197	1.13E-63	*SUV39H1*	2.5951299	6.28E-24
MASTL	2.9784027	1.73E-55	*APOLD1*	2.5948861	1.74E-48
C5orf34	2.8860884	9.63E-36	*AC091057.6*	2.5932367	1.10E-36
PTTG1	2.8845071	2.25E-61	*AL031777.3*	2.578981	1.20E-41
CCDC150	2.8789843	7.70E-36	*CHEK2*	2.5584069	7.84E-39
ZNF888	2.877826	3.53E-07	*UBE2T*	2.5574893	5.49E-64
CAMP	2.8753721	0.004483	*AC002310.2*	2.5539193	7.90E-12
HIST1H2AB	2.859723	3.08E-29	*HMGB2*	2.5508806	5.31E-71
ATP12A	2.8572891	0.0034743	*MYBPC1*	2.5503448	6.71E-05
TBC1D31	2.84845	3.51E-48	*SH3BGR*	2.5481319	5.32E-17
BRCA2	2.8448001	9.34E-56	*RDM1*	2.528142	4.93E-14
FBXO5	2.8396253	3.30E-47	*REXO5*	2.5203376	6.79E-33
FAM83D	2.8332907	1.69E-41	*MFAP4*	2.5182157	1.13E-23
AFAP1L1	2.8297868	1.77E-22	*HIST1H1D*	2.5039846	6.11E-34
AL358075.2	2.8241241	0.0151267	*CENPN*	2.5012945	1.73E-64
KCNG1	2.8239567	1.10E-10	*AC016205.1*	2.48695	1.36E-25
AP000842.3	2.8225381	0.0036171	*MYOCD*	2.4861614	1.27E-05
EDA2R	2.7982424	0.0117953	*MIS18BP1*	2.4831347	2.18E-63
TRAIP	2.7980001	1.45E-47	*PLK4*	2.4797296	2.05E-34
CCDC18	2.7893582	5.36E-50	*ZNF385B*	2.4739712	2.16E-07
HIST1H3F	2.7831125	1.31E-38	*CDCA5*	2.466339	2.26E-59
ZNF850	2.7673943	1.96E-51	*TNFAIP8L1*	2.4615085	2.48E-22
AC073529.1	2.7142572	9.48E-11	*TRIB3*	2.4402874	3.51E-14
INHBE	2.7127264	6.46E-41	*EMC9*	2.4399692	6.10E-40
HIST1H2AI	2.712575	2.12E-39	*ZNF492*	2.4226859	2.11E-30
MITF	2.6995047	0.0006969	*AC024132.3*	2.4226258	0.0014585
PKMYT1	2.6978982	3.63E-52	*HMX2*	2.4115536	1.01E-13
NAV2-AS3	2.6946158	2.30E-06	*AC137834.2*	2.4030921	9.63E-06
BLM	2.682452	4.12E-46	*AC010173.1*	2.3911885	0.0050118
KCNK5	2.6691926	0.0129141	*HIST2H2AA4*	2.3884286	1.19E-05

RAD51AP1	2.3869144	9.08E-51	*ADRB2*	2.1066158	6.04E-11
TUBA1C	2.384632	1.00E-37	*HIST2H4B*	2.1038656	8.11E-11
DNA2	2.3731907	7.24E-34	*HIST1H2AM*	2.098125	6.26E-18
MKX	2.3626402	5.91E-11	*EXPH5*	2.093912	9.94E-16
AC084782.1	2.3534387	6.09E-06	*TBXT*	2.0914909	2.91E-06
TTF2	2.3430125	1.91E-50	*CEP135*	2.089283	1.58E-29
AL035681.1	2.3281676	8.62E-16	*YBX2*	2.0826717	3.47E-12
SPDL1	2.3212763	9.46E-45	*MNS1*	2.0723741	2.83E-25
RFESD	2.3170976	3.34E-06	*AUNIP*	2.0587318	6.87E-12
CD274	2.3017104	5.06E-05	*COLCA2*	2.0535646	7.11E-06
ANP32E	2.2971876	7.04E-61	*FAM122B*	2.0483742	1.67E-39
SLCO4A1	2.2962845	0.024956	*NDOR1*	2.0483047	0.0067179
CKAP5	2.2804401	1.44E-36	*FANCI*	2.0457189	1.71E-32
ALDH1A1	2.2794902	3.11E-06	*FANCD2*	2.0454057	6.20E-38
TRMU	2.2683742	5.97E-33	*NEURL1B*	2.0358549	8.01E-34
HAUS8	2.2656267	1.89E-38	*DMGDH*	2.0306923	1.05E-14
HIST1H2BL	2.2595238	1.61E-24	*HUNK*	2.0203707	2.61E-32
TEDC1	2.2558693	5.28E-25	*SLC28A3*	2.017966	1.34E-05
GDF15	2.244066	8.07E-09	*ZNF165*	2.0122925	1.38E-10
AL359513.1	2.2410413	1.13E-24	*C17orf99*	2.0079447	0.0001183
HIST1H3J	2.2318193	0.0009408	*CALCB*	2.0073406	1.46E-09
CCNF	2.2127843	2.15E-45	*AL603756.1*	1.9997535	7.11E-05
AC008543.1	2.2092885	5.99E-16	*GREM1*	1.9979615	7.47E-12
C21orf58	2.2071454	1.52E-44	*MGME1*	1.9967037	2.42E-35
NEMP1	2.203393	6.24E-35	*CPED1*	1.9966335	0.0092842
APOBEC3B	2.2017153	1.15E-13	*LINC01572*	1.9940758	4.97E-12
CD58	2.2013693	9.09E-10	*CCDC148*	1.9884195	3.35E-08
TNFRSF9	2.1996355	0.0056716	*GEN1*	1.979557	4.97E-27
ZNF695	2.1923549	1.27E-27	*AF001548.2*	1.9784762	3.59E-06
BFSP1	2.1916694	0.0011786	*CENPM*	1.9695828	1.22E-44
GLI1	2.1852569	4.21E-09	*NCAPG2*	1.9625507	2.09E-33
PART1	2.17132	0.0002081	*CDC25B*	1.9595448	3.44E-28
CENPO	2.1601491	1.15E-30	*SRGAP2*	1.958908	3.00E-31
C18orf54	2.1583354	3.65E-32	*AC026401.3*	1.9481822	4.12E-20
LIN54	2.1458688	3.42E-22	*NDE1*	1.9468004	7.15E-23
MYH11	2.1372986	2.81E-13	*TNFSF15*	1.9445182	0.0007216
ARHGAP19	2.1335049	9.22E-17	*AP001347.1*	1.9417141	2.66E-13
ORC6	2.132758	1.30E-48	*AC127002.1*	1.9411406	0.0068138
HIST1H3I	2.1245088	6.54E-15	*AC025176.1*	1.9388362	0.0003782

ZNF90	1.9300292	7.01E-18	*ZSCAN31*	1.7642188	2.79E-09
CEP128	1.9284282	1.66E-21	*ERI2*	1.7608543	1.03E-14
CEP70	1.9237773	5.26E-21	*SRGAP2B*	1.7594256	1.76E-31
PKN3	1.9197294	3.10E-06	*SLC4A8*	1.7579073	4.85E-17
PRSS1	1.9191259	7.81E-13	*CEP295*	1.7523057	1.97E-21
CKS2	1.909039	2.24E-36	*RANGAP1*	1.7498934	3.81E-19
DNMT3B	1.9009748	4.85E-11	*CCDC15*	1.7443051	3.22E-27
ZWINT	1.900116	1.21E-46	*E2F7*	1.7414593	5.96E-29
XRCC2	1.8935274	1.09E-29	*GOT1*	1.7353013	2.17E-15
MTFR2	1.8872523	1.49E-17	*ERLIN1*	1.7326519	7.18E-06
PLIN4	1.8773885	5.61E-05	*FANCB*	1.7272571	8.55E-31
BEST1	1.8711376	0.0005217	*CABYR*	1.7226567	5.55E-07
SRGAP2C	1.8651064	1.85E-32	*HIST1H1C*	1.7216396	2.77E-32
BRD8	1.8632409	1.03E-30	*TRIM66*	1.7186448	1.00E-11
SYNE3	1.8597698	1.32E-17	*LMO7*	1.7178931	3.83E-19
TLR1	1.8536073	0.0099264	*RAD54L*	1.7125264	7.02E-18
GALNT17	1.8420316	4.14E-09	*CHRNA10*	1.7107739	0.0016312
TK1	1.8389556	1.76E-39	*LRR1*	1.7094886	3.27E-24
LAMP3	1.8357881	1.04E-09	*LIN9*	1.7084908	1.07E-19
IRF8	1.8319562	4.60E-06	*RGS5*	1.7071791	0.0005095
AC091980.2	1.8312731	0.0020279	*TNFRSF13C*	1.7027572	0.0002714
ARL6IP1	1.8275197	1.25E-25	*SLC7A3*	1.7007274	0.0421461
ONECUT1	1.8267128	0.0227885	*NCAPD3*	1.6982801	7.95E-30
ATAD2	1.8246676	4.11E-26	*CENPL*	1.6968032	1.87E-29
BRIP1	1.8149104	1.07E-22	*CDC25A*	1.6938356	2.75E-25
SNAI1	1.8131791	8.53E-11	*ACBD7*	1.6911801	8.61E-10
MCM10	1.8111693	6.45E-22	*DBF4B*	1.6871768	2.13E-14
LINC01224	1.8108017	1.87E-09	*OSBPL3*	1.6844691	4.85E-06
TENM2	1.8092832	1.76E-13	*UBE2S*	1.6839105	2.47E-51
CEP192	1.7948659	6.36E-31	*RHNO1*	1.6747569	1.54E-28
TAF5	1.7886112	8.54E-16	*IFFO1*	1.6727116	0.0477276
GPC5-AS1	1.7855017	2.88E-07	*GPSM2*	1.6681021	5.61E-29
ARMC4	1.7831922	6.77E-06	*ZNF536*	1.6668294	2.97E-19
UBL7-AS1	1.7789175	1.60E-16	*BORCS8-MEF2B*	1.6667218	9.16E-06
HIST1H2BC	1.7787277	3.15E-25	*LMNB2*	1.6653158	4.94E-31
C1orf112	1.7769769	1.24E-24	*DARS2*	1.6641777	7.48E-12
TLX3	1.775488	7.73E-21	*NUP37*	1.6527081	4.62E-16
ENDOG	1.7752781	2.64E-32	*SCLT1*	1.6516901	2.03E-20
HIST1H2AG	1.766172	1.12E-34	*ELMO1*	1.6513804	0.0043414

KIF22	1.6488251	8.48E-39	*DZIP1L*	1.5310547	2.42E-06
CENPJ	1.6429168	2.07E-24	*AC011447.3*	1.5305084	4.15E-26
TESMIN	1.6405399	0.0139444	*CRIP3*	1.5274395	0.041572
RNF227	1.6383333	1.67E-06	*BRCA1*	1.5263713	5.04E-22
CCNG2	1.6308605	8.75E-20	*FAAP24*	1.5256023	2.54E-13
TRIM59	1.630771	1.96E-21	*ASF1B*	1.5212625	4.07E-37
CDCA4	1.6291183	9.68E-24	*SCGB1B2P*	1.5208498	0.0005111
RGS3	1.6283826	2.88E-19	*IKZF3*	1.5207152	4.51E-07
COL2A1	1.6273524	2.75E-08	*EMX2*	1.519703	6.31E-15
PKP4	1.6271144	1.19E-24	*DRAM1*	1.5190436	0.0087117
CACNG6	1.6134864	1.65E-06	*DDIT4*	1.5170601	9.00E-15
DTYMK	1.609825	4.64E-39	*SLC7A11*	1.5147759	7.70E-09
GTPBP2	1.6024626	3.76E-17	*ZWILCH*	1.514662	3.05E-15
FAM111A-DT	1.6023229	1.28E-05	*PLXNC1*	1.5052584	1.96E-08
CPM	1.6017554	6.13E-09	*SLC29A3*	1.5046071	7.18E-05
CHTF18	1.5979044	1.59E-15	*HSD17B11*	1.5019264	0.0003157
VRK1	1.593427	3.44E-25	*H2AFX*	1.4986767	1.42E-38
JAM2	1.5922238	1.84E-07	*HMGB3*	1.4952844	1.57E-33
PRSS2	1.5876261	0.0038013	*PHF19*	1.4944662	1.14E-18
FOXB1	1.5858533	1.40E-08	*HMGB1*	1.4944148	1.50E-57
CENPU	1.5843325	3.78E-32	*HIST1H2BO*	1.4910548	2.34E-07
ODF2	1.5771627	1.75E-13	*LMNB1*	1.4870662	4.65E-32
RIBC2	1.5757573	1.36E-13	*ATAD5*	1.4807794	2.42E-18
CLGN	1.5734143	8.28E-14	*METTL4*	1.478934	2.42E-21
MTBP	1.5657875	3.67E-11	*MFGE8*	1.4698758	0.0028706
ZMYM1	1.5636264	5.56E-17	*ZNF436*	1.469679	3.62E-05
HIST1H4B	1.5608752	3.45E-06	*RAD21*	1.4667779	4.83E-49
AC026250.1	1.55888	0.0001736	*MZT1*	1.4634867	1.59E-28
SCML2	1.5560689	7.14E-16	*KIAA0586*	1.4585041	1.41E-16
HIST1H2AH	1.5506655	4.56E-15	*ACSL1*	1.4568369	3.20E-08
BDKRB2	1.5469471	1.32E-05	*AC022075.1*	1.4557556	0.008618
SKA2	1.54625	1.56E-31	*POC5*	1.4551035	6.25E-13
UBALD2	1.5462105	4.89E-28	*SEPHS1*	1.4545741	3.66E-18
KPNA2	1.5455483	3.64E-27	*MYLK*	1.4491059	1.77E-05
MLXIPL	1.539518	4.02E-07	*TEDC2*	1.4467635	5.05E-11
LBR	1.53807	5.98E-25	*PPM1E*	1.4464705	4.37E-16
TMEM79	1.5361954	4.16E-08	*AC120114.1*	1.4453771	0.0107209
HRASLS	1.5344309	0.0001268	*TSPAN19*	1.4442281	0.0084596
SMC2	1.5334488	8.33E-39	*FAM122C*	1.4439841	6.62E-10

COL27A1	1.4438817	0.003452	*RGS16*	1.327359	1.13E-12
STK17B	1.4389875	9.65E-13	*TMPO*	1.3269768	8.57E-42
KBTBD2	1.4379058	1.69E-16	*WDSUB1*	1.3246032	1.71E-06
RNFT2	1.437249	0.0004179	*CDH13*	1.3239833	0.0002462
CEP152	1.4346025	7.87E-21	*TUBA1B*	1.3230968	1.21E-35
PAX6	1.4327163	8.20E-15	*KCTD9*	1.3217471	1.47E-13
HIST1H3H	1.4315404	1.35E-21	*RCC1*	1.3210326	8.91E-10
ARHGAP33	1.4295722	1.07E-17	*AL035461.2*	1.318358	1.25E-06
ULBP1	1.4217592	7.43E-07	*SCML1*	1.3159081	1.33E-15
ARHGEF2	1.4195816	1.33E-22	*PILRA*	1.3141287	0.0090787
DDX39A	1.414067	5.69E-33	*MID1*	1.313368	2.14E-13
G2E3	1.4109439	2.95E-13	*MPHOSPH9*	1.3098101	9.50E-20
CENPQ	1.4092251	9.73E-21	*BARD1*	1.3074117	2.02E-13
ESRP1	1.4073015	3.65E-22	*NFKBID*	1.3041922	6.54E-13
IL23A	1.4038359	9.66E-10	*GSN-AS1*	1.2993153	0.0181179
RNF26	1.3980532	2.07E-09	*FANCM*	1.2988513	2.40E-09
RECQL4	1.3953941	2.45E-14	*NUCKS1*	1.2927953	7.17E-47
ATF5	1.3935044	1.09E-14	*SEL1L*	1.2909929	4.52E-10
AKNA	1.3913664	2.60E-08	*CALM2*	1.2848655	3.90E-45
RIPPLY3	1.3823086	5.87E-20	*CCDC34*	1.2842716	5.86E-24
ILVBL	1.3811108	0.0002887	*ARID5B*	1.2840144	3.08E-07
MAGI1	1.3761555	1.61E-05	*CBR3*	1.2827917	4.52E-10
AC091563.1	1.3733725	0.005264	*DTL*	1.2825848	1.06E-13
ZNF107	1.3732026	1.00E-14	*CDK5RAP2*	1.2817047	6.07E-31
SUV39H2	1.3713402	6.04E-06	*FANCG*	1.2812611	4.86E-13
FAM43A	1.3684755	3.13E-11	*GPR83*	1.2797138	0.0018272
BUB3	1.3669742	5.95E-32	*CEP85*	1.2796756	1.02E-06
SP8	1.3643725	1.99E-13	*EPS8*	1.271524	6.56E-15
TDP1	1.3602445	1.43E-10	*ADD2*	1.2688966	9.64E-12
ZNF257	1.3580164	1.67E-10	*FAM216A*	1.2662118	0.0431584
DDIT3	1.3573971	1.88E-09	*HIST1H3A*	1.2634294	1.83E-07
CENPK	1.3544693	2.79E-21	*TMEFF2*	1.2627222	0.000152
RFC3	1.3511568	4.88E-21	*C2orf69*	1.260511	1.31E-13
RAD54B	1.3499638	1.27E-08	*PARP2*	1.2595234	1.26E-12
COL4A2	1.3472805	8.73E-06	*POLA2*	1.2582977	4.50E-13
ZGRF1	1.3446544	9.36E-10	*IFT122*	1.2572049	1.07E-10
CRISPLD2	1.3417984	9.97E-09	*CMC2*	1.2562246	1.24E-32
VANGL1	1.3416133	4.86E-10	*GINS4*	1.2539604	6.79E-10
FBXO48	1.3292761	4.35E-06	*NEDD4*	1.2525744	0.00305

PLEKHG3	1.2438732	0.0011531	*ZNF726*	1.1926468	3.93E-12
GABPB1	1.2437389	1.29E-11	*LINC01686*	1.1902179	0.0003429
PCNT	1.2413372	4.46E-15	*ADPRHL1*	1.1883192	2.41E-09
ZNF501	1.2407576	7.71E-05	*TMEM60*	1.1853232	3.17E-06
GIN1	1.2407157	0.0049981	*CDC23*	1.184612	8.68E-11
CNTRL	1.2395091	3.37E-14	*11-3*	1.1816917	0.0003025
AL121944.1	1.2385084	5.37E-08	*FAM3B*	1.1778069	0.0006999
MCAM	1.2384673	2.41E-10	*METTL7A*	1.1734214	3.21E-06
BLOC1S5	1.237624	2.47E-06	*EMX2OS*	1.1704697	0.0034259
GTF2A1	1.2359249	6.41E-13	*TMPO-AS1*	1.1691087	1.74E-09
RHEBL1	1.235497	0.0006635	*SAPCD2*	1.1679306	2.64E-14
CWF19L1	1.2329854	0.0026353	*ZNF724*	1.1678334	2.53E-16
UACA	1.2308135	3.89E-10	*RFWD3*	1.167006	4.81E-17
LETM2	1.2300556	0.0008284	*SRBD1*	1.1639273	6.53E-06
AL031666.1	1.2288135	0.0037054	*TRIM45*	1.1635171	2.17E-06
SMOC1	1.2279949	6.38E-05	*CDK19*	1.1621629	5.71E-19
SIPA1L2	1.226701	1.64E-08	*BACH2*	1.1603077	1.78E-11
P4HA2	1.2266255	0.0368832	*RCCD1*	1.1580525	2.05E-06
CEBPG	1.2259653	3.33E-17	*PAK4*	1.1570154	4.96E-12
VEGFA	1.2231625	1.48E-08	*INSM1*	1.155272	4.37E-23
PKIB	1.2227805	0.0002054	*SAE1*	1.1550943	1.76E-16
EFCAB11	1.2227324	3.36E-11	*ZNF273*	1.1526303	1.51E-09
MYO5C	1.2227027	0.0061113	*JADE1*	1.1493092	5.17E-13
ADM	1.2194342	1.47E-07	*NLRP11*	1.147212	0.0361397
CSE1L	1.2190804	1.10E-16	*ZCCHC8*	1.147196	2.50E-11
RBM15	1.2174109	1.04E-12	*RHEB*	1.1467159	7.48E-36
GINS1	1.2172836	9.20E-10	*RAI2*	1.1466696	0.000131
E2F2	1.2149666	5.31E-07	*FBXL18*	1.1461014	0.0070042
HIST1H2BN	1.2119821	1.57E-16	*ZNF519*	1.1455398	6.15E-12
HMGN5	1.2106166	3.18E-07	*OSER1-DT*	1.1434952	0.0019831
AP000251.1	1.2098049	0.0015281	*PTX3*	1.1433695	0.0001562
CCDC77	1.2038296	1.18E-15	*ELOVL2*	1.1425501	2.14E-07
FAM129A	1.2035625	1.40E-07	*NUP35*	1.1416444	2.13E-06
ZNF85	1.1959175	4.24E-13	*PCLAF*	1.1406779	4.50E-15
XPOT	1.195886	1.41E-12	*MCM8*	1.1389962	8.47E-11
PIGK	1.1953501	1.43E-07	*RGS7BP*	1.1378634	5.97E-05
TUBB4B	1.1948246	7.57E-24	*AC010327.4*	1.1372715	0.0002442
NAV2	1.1945463	7.80E-10	*NEFH*	1.1353189	5.65E-07
LARP6	1.1930588	1.25E-10	*SPATA5*	1.1334159	0.0004275

CSTF1	1.1311841	1.92E-06	*FAM110A*	1.0704116	3.26E-09
RRM1	1.1291228	4.69E-16	*TRIM69*	1.0702012	1.26E-05
HIST1H1E	1.1287885	1.13E-13	*AC004943.2*	1.0690377	5.83E-05
GTF2E1	1.1244069	0.0374935	*HIST1H4D*	1.0688157	0.002047
SYCE2	1.1243616	0.0018721	*SDC3*	1.0681547	0.0001841
BTBD10	1.124263	1.54E-08	*HIST1H2BH*	1.0646736	8.70E-36
RASSF1	1.1135532	4.83E-09	*SGTB*	1.0606492	2.19E-07
PSAT1	1.1127388	4.01E-10	*EXO5*	1.0589094	7.51E-05
TGIF1	1.1092551	1.69E-06	*CPOX*	1.0579626	0.0001542
SPA17	1.1086435	1.51E-08	*ZNF597*	1.0556241	0.0341443
FHOD3	1.1083653	8.38E-07	*NCAM1*	1.0547153	1.49E-11
AC097534.2	1.1080576	8.21E-09	*ZNF700*	1.0526137	2.42E-06
ZNF530	1.1073653	7.28E-06	*XYLT1*	1.0512642	5.63E-05
MYBL2	1.1059456	2.33E-10	*DCLRE1C*	1.0510736	2.12E-11
AL355488.1	1.1057509	0.012894	*ERAP2*	1.0494479	1.78E-05
WDR53	1.1044584	5.67E-05	*CCDC151*	1.0478443	0.0070797
TUBB	1.1043589	1.58E-31	*ZNF823*	1.0464451	2.30E-05
AC108860.2	1.1029544	0.0021655	*POU3F2*	1.0462648	3.49E-14
DZIP3	1.1001745	1.19E-07	*MMD*	1.0455923	0.0104486
CALN1	1.0986039	0.0205749	*PIGN*	1.045402	0.000133
PDIK1L	1.0954172	0.0328771	*DYRK4*	1.0445689	4.26E-05
DHRS7B	1.0937935	0.0014659	*NUP205*	1.0444334	2.41E-06
TLNRD1	1.0902222	2.44E-07	*TGFB2*	1.0422486	7.74E-08
CNTROB	1.089365	1.14E-08	*STAC3*	1.0393668	0.0473993
C12orf4	1.0867824	3.77E-08	*CTCF*	1.0393078	1.98E-18
NDC1	1.0855439	9.14E-07	*GPR19*	1.0391462	3.28E-05
G0S2	1.0851638	0.003996	*FZR1*	1.0370567	4.18E-09
THAP10	1.0849835	3.58E-07	*CEP57L1*	1.0359314	5.41E-08
DMRTA2	1.0848722	3.31E-07	*MEIS2*	1.0357553	0.0004462
MIS18A	1.0846624	1.45E-11	*AL441992.1*	1.0347236	9.83E-05
C12orf65	1.0820407	1.76E-12	*NEDD1*	1.0346724	1.55E-06
CENPH	1.081905	6.39E-16	*SREBF1*	1.0343517	6.19E-06
CFLAR	1.0770411	3.19E-09	*SUN2*	1.0335255	1.02E-09
PPP2R3B	1.0766123	2.44E-12	*GADD45A*	1.0322262	5.29E-08
RTP1	1.074596	5.85E-05	*RBL1*	1.0319837	2.40E-05
ZSCAN16	1.0745659	5.92E-06	*DAND5*	1.0307626	3.52E-06
AC008397.2	1.0725336	0.0030807	*CARD8*	1.0303262	2.06E-08
PYCARD-AS1	1.0710832	0.0113309	*AC130324.3*	1.0301408	0.0024083
HACD2	1.0706238	3.84E-11	*KMT5A*	1.0285262	1.44E-11

CBX4	1.0254877	0.0027853
TMEM237	1.0245935	2.61E-07
GINS3	1.0217518	2.07E-06
MSX1	1.0198884	5.31E-09
CDKN2B-AS1	1.0193691	9.20E-10
IKBIP	1.0170621	1.64E-06
SPTSSB	1.0169803	0.0062631
DSCC1	1.0168659	6.81E-13
SEPSECS-AS1	1.0145634	7.54E-06
CMYA5	1.0144505	0.0222635
HIST1H2AE	1.0140898	6.93E-15
CDKN1B	1.0136755	1.31E-19
HPS1	1.0116263	0.0024177
ZNF93	1.009887	1.69E-12
ZNF682	1.005663	1.96E-10
TRAF2	1.0040022	0.0007203
TATDN3	1.0015174	0.000263
PCK2	1.0002457	1.15E-05

Supplemental Table 3. Gene lists which were significantly downregulated (A) or upregulated (B) after KIFC1 knockdown.

A

Gene	log2FoldChange	*p* value
KIFC1	-3.87	0.003
BCYRN1	-3.089	0.001
NPEPPSP1	-3.005	<0.001
ARL17B	-2.856	0.027
FLOT1	-2.825	0.025
CWC25	-2.729	0.001
HRAS	-2.545	0.007
GTPBP6	-2.487	<0.001
NAIP	-2.443	0.008
SMN1	-2.385	0.008
UBR7	-2.158	<0.001
PIP4K2B	-2.152	<0.001
NBL1	-1.984	0.049
PLPPR1	-1.897	0.038
LOC101928882	-1.87	0.008
LOC100289333	-1.65	0.05
GOLGA6L9	-1.538	0.013
LOC441081	-1.383	0.045
PMM1	-1.323	<0.001
TNFSF15	-1.31	0.017
LOC101928596	-1.31	<0.001
LRP4-AS1	-1.285	0.025
MEIG1	-1.241	0.047
GLYATL1	-1.232	0.049
DNAAF5	-1.23	<0.001
SEMA5B	-1.211	0.013
KIZ-AS1	-1.21	0.013
TMEFF2	-1.177	<0.001
CLCN5	-1.144	<0.001
ADAM10	-1.115	<0.001
NALCN-AS1	-1.083	0.046
INTS4P2	-1.076	0.011
KLRG1	-1.037	0.019
CPEB4	-1.03	<0.001
PNMA8B	-1.024	0.022
PCDHGA1	-1.011	0.048
MEX3A	-1	<0.001
TCP11L1	-0.994	<0.001
APCDD1	-0.989	<0.001
SNCB	-0.981	0.005
DENND10	-0.973	<0.001
PCDH12	-0.973	0.042
LOC101929519	-0.919	0.026
ZNF271P	-0.914	<0.001
CEP170	-0.911	0.003
ATP1B1	-0.902	<0.001
IFNGR2	-0.893	0.005
BLACAT1	-0.891	0.019
CD2AP	-0.891	<0.001
MIR4517	-0.887	0.049
PPARD	-0.868	0
STXBP6	-0.861	0.002
TENT4B	-0.852	<0.001
GNB1L	-0.848	0.005
LOC105376780	-0.847	0.045
CMTM1	-0.839	0.034
ZDHHC12-DT	-0.839	0.046
SPATA46	-0.838	0.025
GNG2	-0.836	<0.001
XKRX	-0.832	0.001
IQSEC3	-0.829	0.009
HSF1	-0.827	<0.001
CTDNEP1	-0.817	<0.001
CCDC92	-0.806	0.017
MRTFB	-0.805	<0.001
ADAMTS20	-0.804	0.02
PPM1F	-0.797	<0.001
ALG2	-0.795	<0.001
SLC6A14	-0.791	0.027
WNT5B	-0.784	<0.001
APOL4	-0.781	0.024

SMC1A	-0.776	<0.001	*PEMT*	-0.652	<0.001
ZXDA	-0.771	0	*KCND3*	-0.651	0.023
PIK3C2B	-0.761	0.002	*CASC9*	-0.648	0.002
PIM1	-0.757	<0.001	*DLGAP3*	-0.647	<0.001
PARP2	-0.754	<0.001	*CMTM4*	-0.645	<0.001
LINC00294	-0.754	<0.001	*ZSWIM6*	-0.645	<0.001
TMEM245	-0.754	<0.001	*PCDHB5*	-0.643	<0.001
MPZL3	-0.744	<0.001	*ITGBL1*	-0.64	0.005
MFAP3	-0.744	<0.001	*FAM227A*	-0.638	0.003
SLC32A1	-0.739	0.03	*TP53RK*	-0.635	<0.001
ANGPT2	-0.735	<0.001	*ZFP36L1*	-0.635	0.044
RUFY1	-0.735	<0.001	*NEK10*	-0.633	0.026
TRAF4	-0.725	<0.001	*ZC3H6*	-0.629	<0.001
CPOX	-0.723	<0.001	*NRIP3*	-0.628	<0.001
RSAD2	-0.722	0.031	*MLLT11*	-0.627	<0.001
FOXP4	-0.719	<0.001	*UGT8*	-0.624	<0.001
RNF38	-0.717	<0.001	*ATP10D*	-0.624	0.001
TGFB2	-0.717	<0.001	*C5*	-0.623	0.009
MXD1	-0.709	<0.001	*NDFIP2*	-0.62	<0.001
SH3PXD2B	-0.708	<0.001	*BCL2L13*	-0.617	<0.001
U2AF1	-0.707	0.008	*KLHL28*	-0.617	0.001
DENND10P1	-0.706	0.046	*DNAH1*	-0.614	0.009
PTPRJ	-0.703	<0.001	*RNF26*	-0.613	<0.001
EP400P1	-0.702	0.008	*CBX4*	-0.612	<0.001
FOXB1	-0.699	0.014	*BRD3*	-0.607	<0.001
SLC40A1	-0.69	0.024	*TNR*	-0.607	0.046
RNF152	-0.69	<0.001	*XKR7*	-0.606	0.004
SUSD1	-0.687	<0.001	*SVBP*	-0.605	0.005
BDNF	-0.681	0.024	*VEPH1*	-0.601	0.042
SGMS1	-0.678	0.001	*RPS6KA2*	-0.601	<0.001
LINC00391	-0.674	0.014	*ST6GALNAC5*	-0.599	<0.001
NBPF1	-0.673	0.005	*ACSL4*	-0.597	<0.001
STARD4-AS1	-0.672	0.019	*SHANK3*	-0.597	<0.001
TMEM30B	-0.672	<0.001	*PLCXD2*	-0.597	0.049
ZDHHC4	-0.672	<0.001	*GALNT16*	-0.594	0.007
MTCL1	-0.669	<0.001	*SLC5A3*	-0.59	0.002
CCN2	-0.665	0.004	*IPP*	-0.589	0.034
CELF5	-0.662	0.009	*PLD1*	-0.587	0.006
RAD1	-0.66	<0.001			

B

Gene	log2FoldChange	*p* value
IGHV3-22	3.956	<0.001
IFI27L1	2.95	<0.001
NTAN1	2.937	0.025
HERC2P10	2.721	0.004
DHRS11	2.334	0.006
RNH1	2.254	<0.001
CACNG2	2.204	0.029
MYL11	2.118	0.008
PBX2	2.041	0.028
CHGA	1.995	0.003
LINC01727	1.957	0.035
RSPH6A	1.956	0.039
GJB2	1.949	0.013
JDP2	1.938	0.003
ZAN	1.922	0.041
AKT3	1.872	0.022
SNORD3A	1.83	0.046
GRAMD1C	1.792	0.009
KRT15	1.77	<0.001
CCND2	1.758	0.01
AXL	1.708	0.046
PDE2A	1.598	0.043
MOAP1	1.567	0.003
LYSET	1.523	0.044
LINC01170	1.512	0.022
KANSL1	1.512	<0.001
LY6E	1.477	0.01
LINC00920	1.477	0.039
KLHL6	1.433	0.028
RBM47	1.352	<0.001
LINC02523	1.315	0.019
GP5	1.305	0.044
BEST1	1.293	0.009
TMC8	1.284	0.046
CD19	1.282	0.042
ASB5	1.267	0.017
TNFRSF18	1.253	0.032
HSPA6	1.236	0.019
TRDN	1.229	0.023
SLCO4A1	1.205	0.05
FER1L6	1.196	0.003
SLC6A4	1.143	0.05
SERF1A	1.137	0.043
MYO7B	1.135	0.006
AWAT1	1.101	0.029
SLC25A18	1.1	0.026
CKM	1.075	0.007
PNOC	1.069	0.02
SYPL1	1.057	<0.001
PLCXD1	1.046	0.001
MUC13	1.038	<0.001
LMLN	1.036	<0.001
CELF2	1.018	<0.001
C20orf204	0.996	0.002
MYT1	0.993	0.03
NMB	0.961	0.022
MT1G	0.946	0.009
SPRYD4	0.944	<0.001
EIF5A2	0.937	0.002
UAP1L1	0.936	<0.001
LDHA	0.932	<0.001
OR51E1	0.928	<0.001
CD3G	0.925	0.021
CRLF1	0.923	0.001
RAB7A	0.918	<0.001
AFM	0.914	0.01
HNF4G	0.908	<0.001
HECW2-AS1	0.902	0.001
MLLT6	0.897	<0.001
HTR1D	0.892	<0.001
MBOAT7	0.889	0.022
SLC6A20	0.884	0.009
SERPINB9	0.877	0.039
RPLP0	0.874	<0.001
CYRIB	0.856	<0.001
YBX3	0.847	<0.001

CLDN18	0.847	<0.001	*RAI2*	0.698	<0.001
LAD1	0.84	<0.001	*FBXO27*	0.697	<0.001
BAIAP3	0.838	0.006	*JADE2*	0.695	<0.001
TRNT	0.836	0.025	*CNGA4*	0.692	0.002
TNFRSF19	0.833	<0.001	*BRD2*	0.69	0.017
HSPB1	0.831	<0.001	*FBXO8*	0.687	<0.001
TAMALIN	0.823	0	*SNORA32*	0.687	0.031
FZD8	0.82	0.019	*SAMD11*	0.687	0.016
NR0B2	0.82	0.015	*CSKMT*	0.686	0.028
HECTD2-AS1	0.819	0.015	*DLL4*	0.686	<0.001
KCNK16	0.815	<0.001	*TESC*	0.685	<0.001
LOC102724594	0.814	0.028	*PRR22*	0.683	0.023
TMEM123	0.809	<0.001	*UBE2D1*	0.682	<0.001
BMPER	0.808	0.019	*TMEM37*	0.68	0.005
SECTM1	0.803	0.036	*ABHD16B*	0.68	0.043
GPR37L1	0.8	0.037	*VPS26A*	0.676	<0.001
H2BC12	0.795	<0.001	*ZNF296*	0.669	0.009
SRPRA	0.795	<0.001	*COL16A1*	0.668	0.011
FBXO33	0.794	<0.001	*SETD7*	0.658	<0.001
C16orf74	0.784	0.019	*CASP8*	0.654	0.047
MPRIP	0.779	<0.001	*MT1X*	0.648	0.006
RHOU	0.776	<0.001	*CEP57*	0.647	<0.001
SQOR	0.775	<0.001	*CBLN2*	0.645	0.002
LOC102724112	0.773	0.009	*CYP1A1*	0.643	0.022
PM20D2	0.771	<0.001	*SMIM20*	0.64	<0.001
TLCD3B	0.771	<0.001	*PDK4*	0.639	0.006
ITPK1	0.769	0.004	*PRRG4*	0.638	0.02
INTS14	0.762	<0.001	*HOXB9*	0.638	0.005
RHPN2	0.746	<0.001	*ATAD3C*	0.638	0.009
VKORC1L1	0.745	<0.001	*TCEAL8*	0.637	<0.001
SELENOW	0.744	<0.001	*LRRC1*	0.635	<0.001
GDF15	0.742	<0.001	*FOXP2*	0.635	<0.001
NFATC2	0.738	0.003	*AHNAK*	0.635	0.012
FJX1	0.735	<0.001	*TPM3*	0.634	<0.001
TMEM243	0.731	<0.001	*GLTP*	0.634	<0.001
GRAMD4	0.719	0.008	*TSPAN1*	0.634	0.002
LINC02418	0.718	0.002	*BAALC*	0.633	<0.001
ERVS71-1	0.714	0.01	*ARHGEF4*	0.633	0.004
GJB1	0.699	<0.001	*HOXB6*	0.628	0.003

STK32A	0.627	0.001
STX1B	0.623	0.001
HDAC1	0.623	<0.001
SLC38A3	0.62	<0.001
NKX2-2	0.613	<0.001
GRAMD2B	0.613	0.001
SON	0.611	<0.001
PRKAA1	0.61	<0.001
TASP1	0.609	0.001
RBFOX1	0.607	0.001
EEF1AKMT3	0.607	0.002
ZNF488	0.605	<0.001
TTK	0.604	<0.001
KCNA2	0.603	0.003
DOK7	0.598	0.008
XPR1	0.597	<0.001
SLC43A1	0.596	<0.001
C2orf88	0.593	0.044
TPM2	0.591	0.028
IGFBP5	0.591	<0.001
CXCL14	0.589	0.001
CREB3L2	0.589	<0.001
AMACR	0.589	0.022
