# Supplementary figures and images for: Targeting KIFC1 to disrupt centrosome clustering and trigger anaphase catastrophe in small-cell lung cancer
Source: JCI Insight. 2026 Apr 8;11(7):e199352. doi: 10.1172/jci.insight.199352 (PMC13134724; doi:10.1172/jci.insight.199352)

Figure 2C

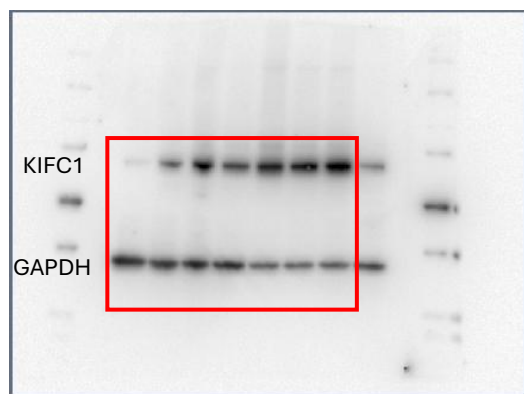

Figure 5C

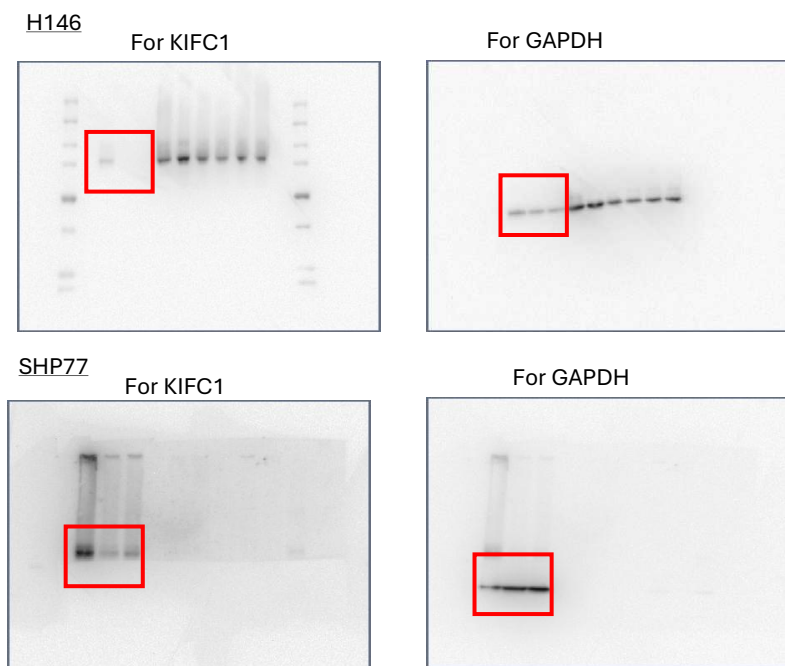

Figure 5E

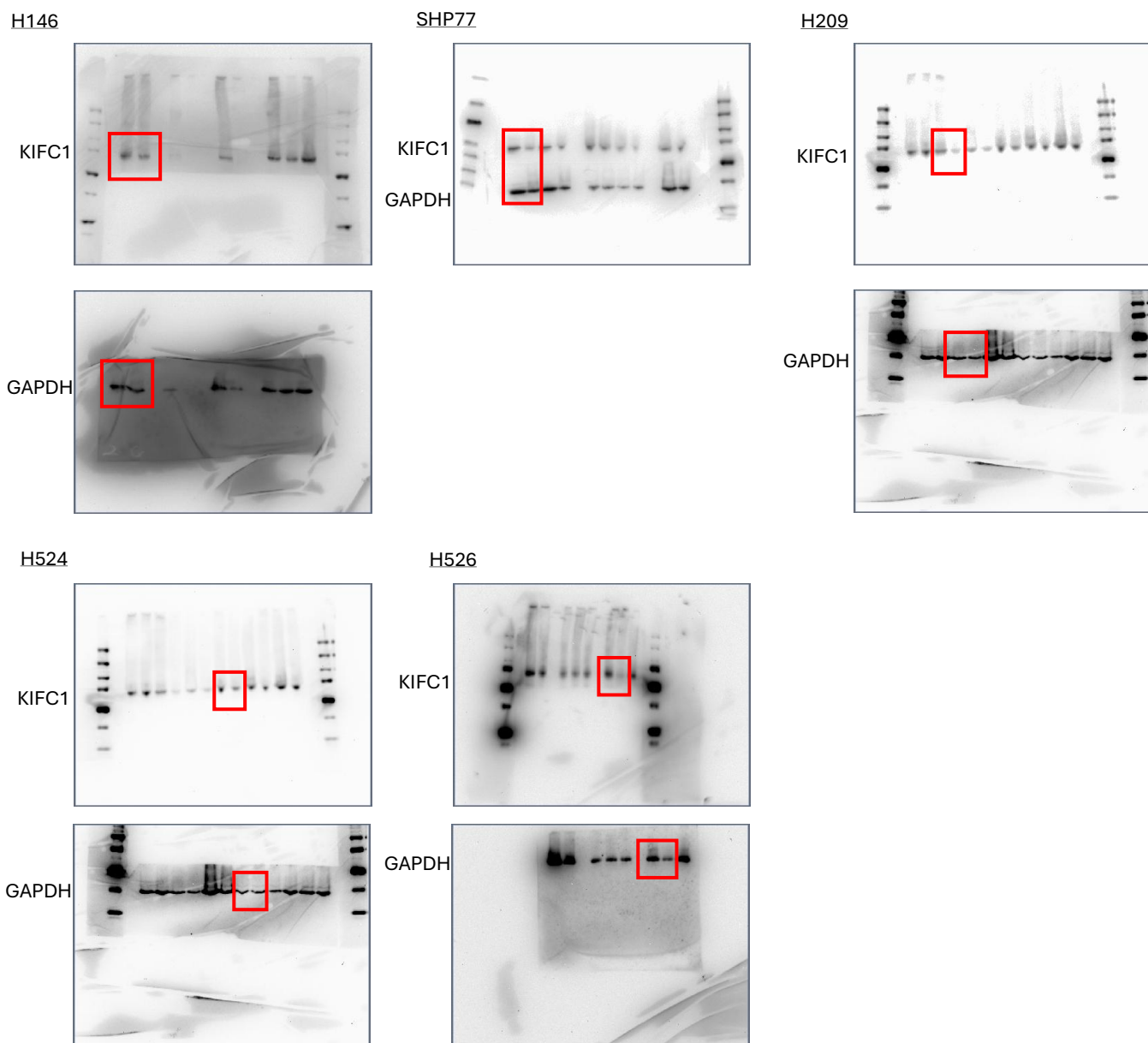

Supplemental Figure 9C

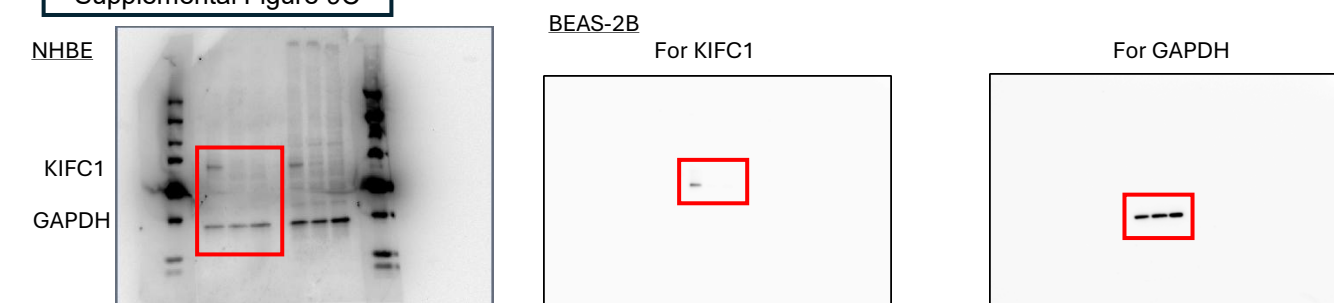

Supplement: Unedited blot and gel images [file jciinsight-11-199352-s117.pdf]
